# Six Metabolism Related mRNAs Predict the Prognosis of Patients With Hepatocellular Carcinoma

**DOI:** 10.3389/fmolb.2021.621232

**Published:** 2021-02-25

**Authors:** Xiwen Wu, Tian Lan, Muqi Li, Junfeng Liu, Xukun Wu, Shunli Shen, Wei Chen, Baogang Peng

**Affiliations:** ^1^ Department of Hepatic Surgery, The First Affiliated Hospital of Sun Yat-sen University, Guangzhou, China; ^2^ Department of Pancreaticobiliary Surgery, The First Affiliated Hospital of Sun Yat-sen University, Guangzhou, China; ^3^ Department of Pathology, The First Affiliated Hospital of Sun Yat-sen University, Guangzhou, China

**Keywords:** metabolism, hepatocellular carcinoma, the cancer genome Atlas, mRNAs, prognostic model

## Abstract

**Background: **Hepatocellular carcinoma (HCC) is one of the most common aggressive solid malignant tumors and current research regards HCC as a type of metabolic disease. This study aims to establish a metabolism-related mRNA signature model for risk assessment and prognosis prediction in HCC patients.

**Methods:** HCC data were obtained from The Cancer Genome Atlas (TCGA), International Cancer Genome Consortium (ICGC) and Gene Enrichment Analysis (GSEA) website. Least absolute shrinkage and selection operator (LASSO) was used to screen out the candidate mRNAs and calculate the risk coefficient to establish the prognosis model. A high-risk group and low-risk group were separated for further study depending on their median risk score. The reliability of the prediction was evaluated in the validation cohort and the whole cohort.

**Results: **A total of 548 differential mRNAs were identified from HCC samples (*n* = 374) and normal controls (*n* = 50), 45 of which were correlated with prognosis. A total of 373 samples met the screening criteria and there were randomly divided into the training cohort (*n* = 186) and the validation cohort (*n* = 187). In the training cohort, six metabolism-related mRNAs were used to construct a prognostic model with a LASSO regression model. Based on the risk model, the overall survival rate of the high-risk cohort was significantly lower than that of the low-risk cohort. The results of a time-ROC curve proved that the risk score (AUC = 0.849) had a higher prognostic value than the pathological grade, clinical stage, age or gender.

**Conclusion:** The model constructed by the six metabolism-related mRNAs has a significant value for survival prediction and can be applied to guide the evaluation of HCC and the designation of clinical therapy.

## Introduction

Hepatocellular carcinoma (HCC) is one of the most aggressive solid malignant tumors with poor prognosis and high mortality. An article on global cancer statistics for 2018 reported that HCC was the third leading cause of cancer-related deaths worldwide (Bray et al., 2018). Only a small number of HCC patients were able to receive curative treatment including hepatectomy resection, transplantation, or ablation at the time of diagnosis. What’s more, even after curative therapy, tumor recurrence occurs in 50–70% of HCC patients within 5 years (Forner et al., 2012). Most of the studies focus on the clinicopathological characteristics of HCC patients to construct prognostic models to predict overall survival (OS) and help to stratify patients’ OS (Xu et al., 2018; Best et al., 2020; Dai et al., 2020).

Recent years have seen a gradual increase in the number of reports on the use of genomics, proteomics and metabolomics technology to study the pathogenesis and tumorigenesis of malignant tumors (Kimhofer et al., 2015; De Stefano et al., 2018; Beyoglu and Idle, 2020). Since metabolic disorder is a key event in the occurrence and development of HCC, HCC has been considered a type of metabolic disease (Piccinin et al., 2019). An increasing number of characteristic metabolic markers related to HCC have been found. Examples include alpha-fetoprotein (AFP) (Yamamoto et al., 2010; Notarpaolo et al., 2017; Pinyol et al., 2019) and PIVKA-II also called des-γ-carboxyprothrombin (DCP) (De Stefano et al., 2018; Loglio et al., 2020). These markers can not only indicate the natural course of a tumor, but also provide evidence for a treatment strategy and an evaluation of the pharmacodynamics of drugs (Zhang and Finn, 2016).

A study has shown that an immune-related prognostic model provides new potential prognostic and therapeutic biomarkers (Wang et al., 2020), whereas few metabolism-related genes were found. The prognostic value of the metabolism-based prognostic model that we constructed (3-years AUC = 0.849) was higher than that of the immune-based prognostic model (3-years ROC = 0.711; 3-years ROC = 0.79) (Wang et al., 2020; Zhang et al., 2020), indicating that the six metabolic mRNAs signature that we conducted had a robust high prognostic value. A recent study identified a four-gene metabolic signature which could efficiently stratify patients’ OS (Liu et al., 2020). However, the prognostic model showed a low diagnostic performance in predicting long-term survival and little is known about the role and mechanisms of these metabolic genes (Liu et al., 2020). In this study we have constructed a gene signature composed of six metabolism-related mRNAs that has significant value in survival prediction and can be used to guide the adjuvant therapy for HCC.

## Materials and Methods

### Data Collection

HCC transcriptome data and corresponding clinical information were downloaded from The Cancer Genome Atlas (TCGA) (https://portal.gdc.cancer.gov/) and International Cancer Genome Consortium (ICGC) (https://icgc.org). GSE22058, GSE25097, GSE45114, GSE62232, GSE121248, GSE76311 and GSE76427 were downloaded from the GEO database to test the expression of six metabolic mRNAs in tumor and normal control tissues. The KEGG data from the GSEA website (https://www.gsea-msigdb.org/) were downloaded for signaling pathway enrichment analysis.

### Samples Cohort

Samples that lacked information on the survival time or survival status were eliminated. The TCGA dataset was randomly divided into a TCGA-training cohort (*n* = 186) and a TCGA-validation cohort (*n* = 187). A metabolic mRNA model of prognosis was established by using the TCGA-training cohort, TCGA-validation cohort and ICGC cohort (*n* = 243) to test the model’s predictive value of the model.

### Screening for Metabolic Gene Signature Related to Prognosis

The R package “*limma*” was used to conduct differential analysis. The screening criteria were |logFC| > 0.5 and a false discovery rate (FDR) < 0.05. “Metabolism” was used as a key word to search all metabolic-related signaling pathways in KEGG. The gene signatures contained in these signaling pathways are considered to be metabolic-related gene signatures. We then intersected all differentially expressed genes (DEGs) with metabolic genes to arrive at all of the metabolic DEGs in HCC. We further screened the metabolic DEGs related to prognosis through Univariate Cox Regression analysis. For the process of analysis we used the R package “survival”. The screening criteria were Hazard ratio > 1.000 and *p*-value < 0.001.

### Construction and Evaluation of a Metabolic mRNAs Prognosis Model

The R package *“glmnet”* was used to build the least absolute shrinkage and selection operator (LASSO) Cox regression model of the metabolic DEGs related to prognosis. First, the risk value of each sample in the TCGA-training cohort was calculated using the following formula:

$$\text{RiskScore}=\overset{n}{\underset{i}{\sum }}\left(\text{expression}\;\text{of}\;mRNAi*Coef_{mRNAi}\right)$$

Then we set the median as the threshold. By comparing the risk value with the threshold, the TCGA-training cohort was divided into a high-risk group and a low-risk group. Second, the same method was used to divide the TCGA-validation cohort and the ICGC-validation cohort into two groups, with a high and low risk respectively. The threshold of each validation cohort was the same as the threshold of the TCGA-training cohort. Third, the R package “survival” was used to test whether there were prognostic differences between the high and low-risk groups in the three cohorts. Fourth, the relationship between clinical features and the risk score was calculated through univariate independent prognostic analysis (UIPA) and multivariate independent prognostic analysis (MIPA). Fifth, the area under the curve (AUC) and the time-dependent Receiver Operating Characteristic (ROC) curves were assessed to evaluate the predictive power of the risk score for survival prediction.

### Building and Validating a Predictive Nomogram

We used the R package “rms” to draw a nomogram that could be used to predict the prognosis of HCC patients. Each clinical indicator corresponded to a score. The patient's clinical characteristics in the nomogram were filled in to get the corresponding score, and the score of each indicator was added to obtain the total score. Variables with no clinical significance were not included in the nomogram. The total score was compared with the 1-, 3-, and 5-years survival probability scale to estimate the patient's possible survival time.

### Validation of Prognostic mRNA Signature Expression

In order to further verify the expression of mRNA signatures in HCC, 7 HCC datasets were downloaded from the Gene Expression Omnibus (GEO) database (https://www.ncbi.nlm.nih.gov/geo/), including GSE22058 (tumor: normal = 100:97), GSE25097 (tumor: normal = 268:243), GSE45114 (tumor: normal = 24:25), GSE62232 (tumor: normal = 81:10), GSE76311 (tumor: normal = 62:59), GSE76427 (tumor: normal = 115:52) and GSE121248 (tumor: normal = 70:37). We retrieved the immunohistochemical staining (IHS) results of the proteins corresponding to these mRNA signatures from the human protein atlas (https://www.proteinatlas.org/, HPA) to obtain the protein expressions. The overall survival was analyzed using GEPIA (http://gepia.cancer-pku.cn/) (Tang et al., 2017).

### Multiple Enrichment Analysis of Risk mRNA Signatures

The original mRNA matrix of three cohorts was divided into a high-risk group and a low-risk group. The two groups were subjected to gene enrichment analysis to obtain the top 5 significantly enriched metabolic signaling pathways. Enrichment analysis was performed using gene-set enrichment analysis software (GSEA_4.0.3). The dataset selected for analysis was KEGG. Each enrichment was carried out for 1,000 cycles, and the R packages “plyr”, “ggplot2”, “grid” and “gridExtra” were used to draw multiple GSEA plots.

### Statistical Analysis

Statistical analyses were performed using SPSS (version 22.0) and R (version 3.6.1 for Mac). The Wilcox test was used in the difference analysis process. If there were multiple lines of a gene in the expression dataset, they were averaged into one line. Pearson χ^2^ test was used to explore random grouping results as appropriate. Except for the correlation coefficient of the model, all numerical values retained three digits after the decimal point. Unless otherwise specified, a *p*-value < 0.05 is considered statistically significant.

## Results

### Screening for 45 DEGs Associated With Poor Prognosis

**FIGURE 1:**
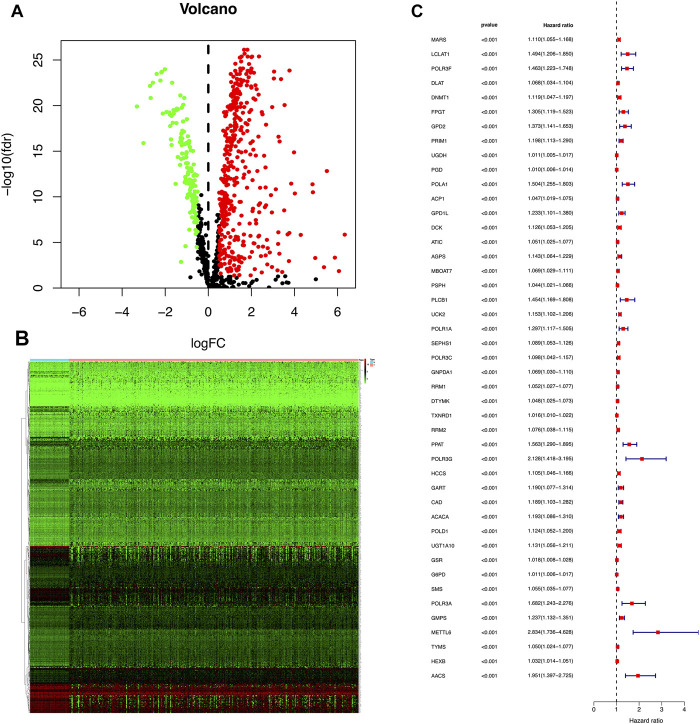
**(A)** A volcano plot was used to show DEGs in TCGA HCC samples. Each point represents an mRNA. Red means a high expression level, green means low expression level, |logFC| > 0.5 and a *p*-value < 0.05 are meaningful. FDR stands for false discovery rate. **(B)** A heatmap was used to show the expression of all differential genes in tumors and normal tissues. **(C)** Red represents high-risk DEGs, whereas green represents low-risk DEGs. The judgment of high and low risk was based on whether the HR value is greater than or equal to 1. There are 45 DEGs in the training cohort that are related to prognosis, and all are high-risk prognosis DEGs and have a *p*-value < 0.001.

Clinical information of the TCGA training cohort and TCGA validation cohort is shown in Table 1. There was no difference in clinical characteristics between the TCGA-training cohort and TCGA-validation cohort in a chi-square test (Table 1). By comparing the HCC tissues in TCGA with the adjacent tissues, we obtained 547 DEGs. Among them, 137 were down-regulated and 410 were up-regulated. Differential expression results were visualized in Figure 1A. The log fold change (log FC) and expression level of all DEGs were in Supplementary Table S1. Subsequently, the 547 DEGs were further screened in the training cohort through survival analysis and 45 DEGs related to prognosis were obtained. These 45 genes were all high-risk mRNAs, and their expression levels were negatively correlated with the prognosis of patients. A heatmap was used to show the expression of all differential genes in tumors and normal tissues in Figure 1B. All of the prognostic DEGs were shown in Figure 1C. Supplementary Table S2 included the Hazard ratio (HR), HR.95L and HR.95H value of each mRNAs.

**TABLE 1:** Clinical information of training cohort and validation cohort.

Clinical data	TCGA-training cohort	TCGA-validation cohort	Total	χ^2^	*p*-value
Gender					
Male	120	133	253		
Female	67	54	121	0.11	1.000
Age					
<60	78	91	169		
≥60	109	95	204	0.154	0.977
Tumor grade					
G1+G2	109	124	233		
G3+G4	74	62	136	0.141	0.970
Survival status					
Alive	120	128	248		
Dead	67	59	126	0.222	1
TNM stage					
I + II	124	136	260		
III + iv	49	41	90	0.183	0.977

TCGA: The Cancer Genome Atlas, Pearson χ^2^ test was used to explore random grouping results as appropriate.

#### Construction of a Prognostic Models Composed of Six mRNA Signatures

**FIGURE 2:**
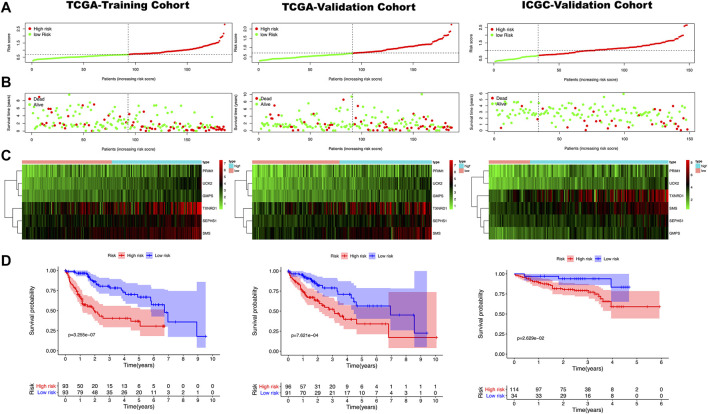
**(A)** The red point represents samples with a high risk score, and the green point represents samples with a low risk score. The risk score on the *x* axis increases from left to right for each sample. **(B)** The red point in the risk plot represents dead samples, and the green point represents survival samples. The left side of the dotted line represents the low-risk group and the right side represents the high group. **(C)** A heatmap shows that the expression level of the six genes used for model construction in three cohorts. Blue and pink represent high and low-risk groups, red and green are used to indicate high and low expression levels. **(D)** A Kaplan Meier curve shows that the overall survival rate of the high-risk group is significantly lower than that of the low-risk group. The red and blue line indicate high and low risk, respectively.

We put the 45 mRNA signatures obtained in the previous step into the training cohort for model construction. Only six mRNAs were ultimately used to construct the model, i.e. primase polypeptide (PRIM1), uridine-cytidine kinase 2 (UCK2), selenophosphate synthetase 1 (SEPHS1), thioredoxin reductase (TXNRD1), spermine synthase (SMS) and granulocyte-monocyte progenitors (GMPS). The correlation coefficient of each mRNA was shown in Supplementary Table S3. The result was the following model: (expression of PRIM1 * 0.0036) + (expression of UCK2 * 0.0498) + (expression of SEPHS11 * 0.0087) + (expression of TXNRD1 * 0.0029)+ (expression of SMS * 0.0109) + (expression of GMPS*0.0207). This formula was used to calculate the risk score of each sample in the training cohort with the medium score 0.692 as the threshold. Then, 93 cases were allocated to high-risk group and 93 cases to the low-risk group. Using the same formula and threshold, we ended up with 96 samples in the high-risk group and 91 samples in the low-risk group in the TCGA-validation cohort, and 114 samples in the high-risk group and 34 samples in the low-risk group in the ICGC-validation cohort. The overall grouping results tended to be average (Figure 2A Supplementary Table S4). The risk curve shown in Figure 2B indicated that as the risk score increased, the number of deaths also increased. The results of the differential analysis between the high and low risk groups showed that the expression levels of these six genes in the high-risk group were higher than in the low-risk group (Figure 2C). Kaplan Meier curve showed that the prognosis of the high-risk group was significantly worse than that of the low-risk group (Figure 2D).

#### The Model Could Predict HCC Patient Survival

**FIGURE 3:**
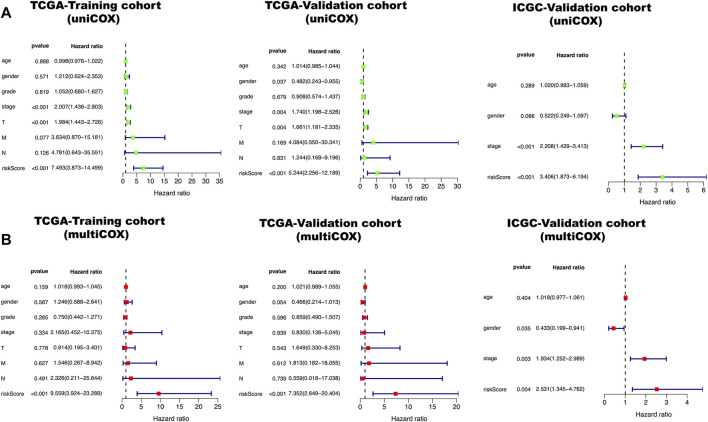
Green represents a univariate independent prognostic analysis (UIPA), red represents a multivariate independent prognostic analysis (MIPA). **(A)** In the UIPA, the TNM stage (*p*-value < 0.001) and risk score (*p*-value < 0.001) can be used as independent prognostic judgment factors in three cohorts. **(B)** The risk score (*p*-value < 0.001) can be used as an indicator of prognostic judgment.

**FIGURE 4:**
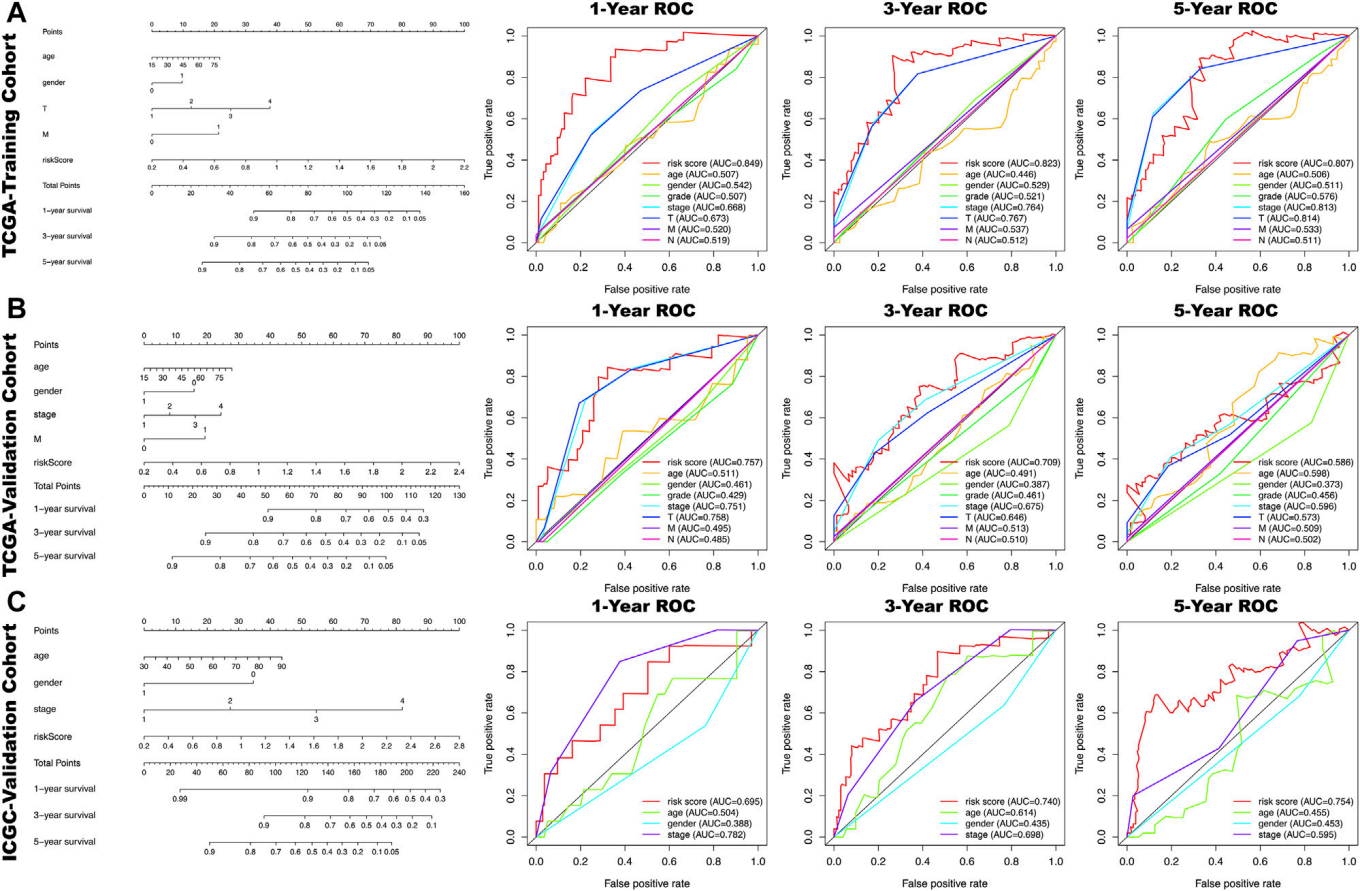
In the nomogram, each clinical indicator and risk score corresponds to a score and the total score corresponds to the patient's 1-, 3- and 5-years survival probability. The time-dependent ROC curves of the risk score, age, gender, grade, stage, T, N, M for 1-,3- and 5- year overall survival (OS) in TCGA-training cohort **(A)**, TCGA-validation cohort **(B)** and ICGA-validation cohort **(C)**.

All clinical characteristics and risk scores were subjected to a univariate independent prognostic analysis (UIPA). The UIPA results showed that TNM stage (*p*-value<0.001) and risk score (*p*-value<0.001) could be used as independent prognostic judgment factors, and the results were validated in the training cohort and the two validation cohorts, respectively (Figure 3A). MIPA analysis showed that only the risk score could be used as an independent prognostic judgment factor (Figure 3B). The Hazard ratio (HR), HR.95L and HR.95H value of each mRNA were listed in Supplementary Table S5. The nomogram used to evaluate the 1-, 3- and 5-years survival rate of patients was shown in Figure 4A. In the results of the ROC curve of the training cohort (Figure 4B), the Area Under Curve (AUC) of the risk score was 0.849 and the value for judging the prognosis far exceeded that of the indicators such as age, gender, pathological grade and TNM stage. In the two validation cohorts, the risk score also performed well (TCGA-AUC = 0.757, ICGC-AUC = 0.695).

### Verification of Expression Levels of Six mRNAs

**FIGURE 5:**
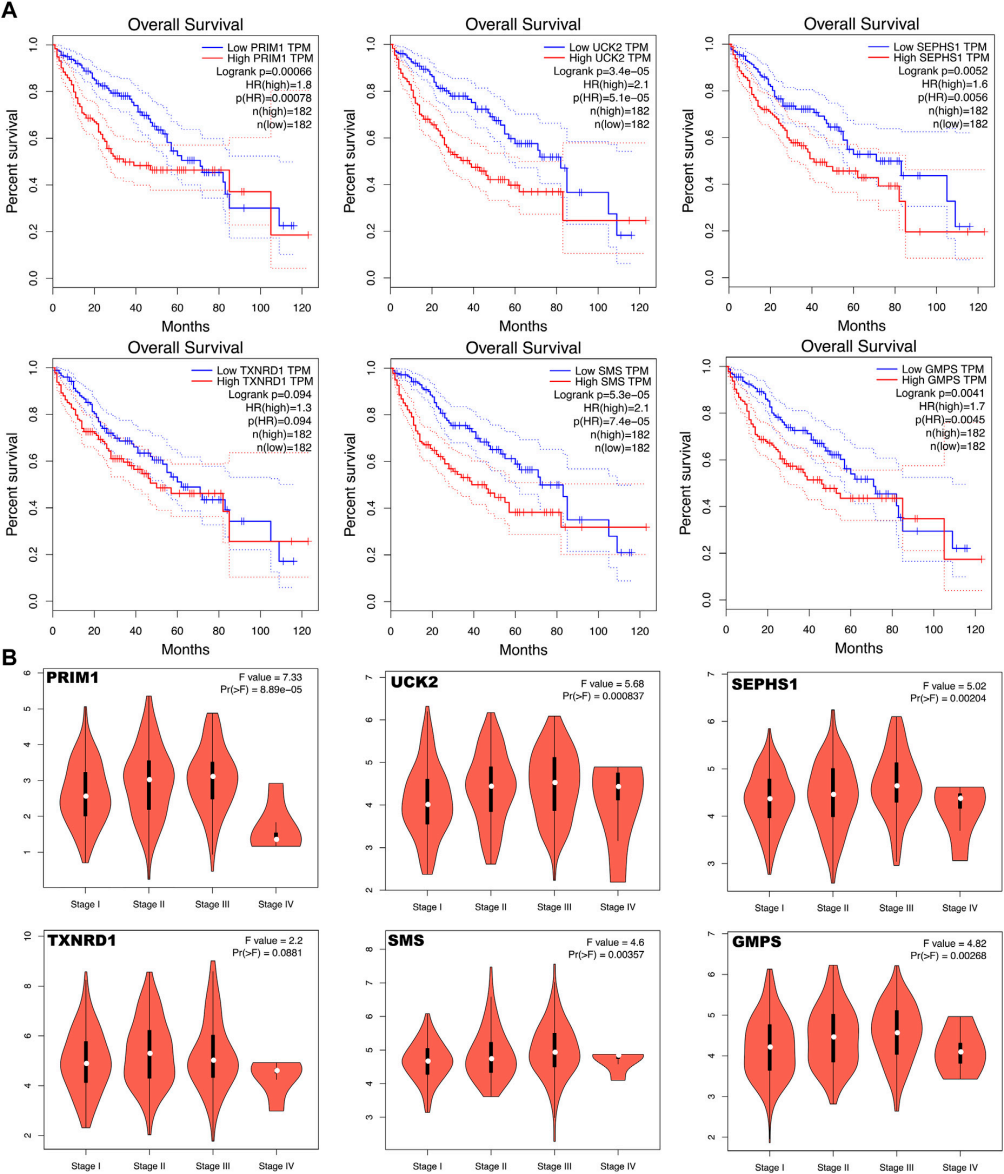
**(A)** Kaplan Meier curve shows that the overall survival rate of the high expression levels of the six mRNA signatures are significantly lower than that of the low expression levels. **(B)** Violin chart shows the relationship between gene expression level and clinical stage.

**FIGURE 6:**
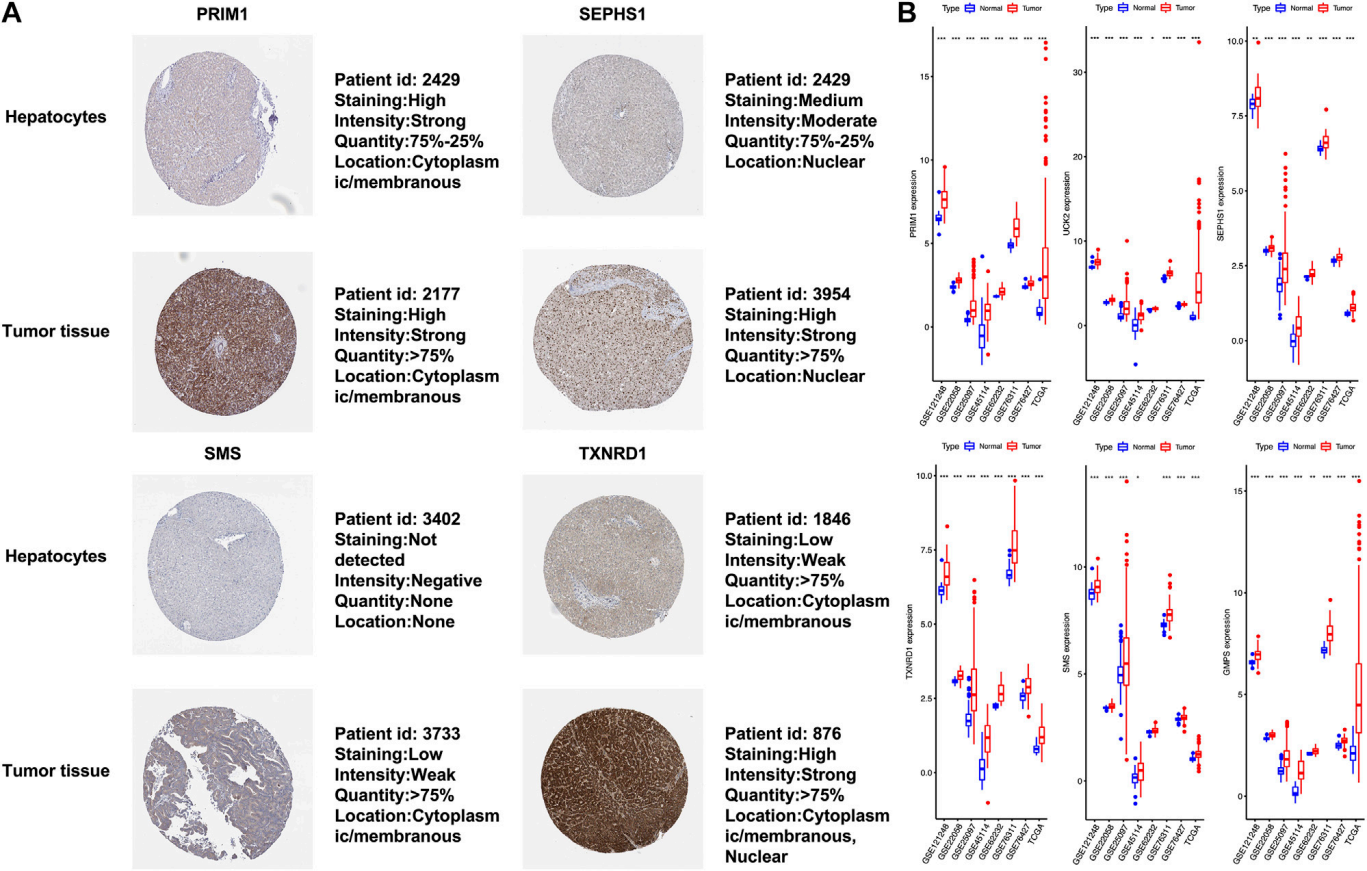
**(A)** In terms of staining, SEPHS1, SMS and TXNRD1 stain more strongly in tumor cells than normal hepatocytes, whereas this is not the case for PRIM1. All four proteins are stained in more than 75% of tumor cells. PRIM1 and SMS are both expressed in the cytoplasm and membrane, SEPHS1 is only expressed in the nuclear fraction. Unlike the other three, TXNRD1 has a wider cell localization, and protein expression has been detected in the cytoplasm, membrane and nuclear fraction. **(B)** The *x*-axis represents each dataset, and the *y*-axis represents mRNA expression. * means *p*-value = 0.01–0.05, ** means *p*-value = 0.001–0.01, and *** means *p*-value < 0.001.

The six mRNAs i.e. PRIM1, UCK2, SEPHS1, TXNRD1, SMS and GMPS, all had higher expression levels in the HCC sample than in normal controls, which was consistent in the eight datasets. What’s more, the samples with high expression levels had lower overall survival rates than the samples with low expression levels in the TCGA dataset (Figure 5A). A violin chart shows the relationship between gene expression level and clinical stage (Figure 5B). The HPA database contained only the immunohistochemical results of PRIM1, SEPHS1, TXNRD1 and SMS. The results showed that the proteins corresponding to four mRNA signatures had high expression level in HCC cells compared with normal hepatocytes (Figure 6A). Except for the *p*-value of SMS in GSM62232, which was larger than 0.05, all other results have a *p*-value < 0.05, which was statistically significant (Figure 6B).

### GSEA Enrichment Analysis Results

**FIGURE 7:**
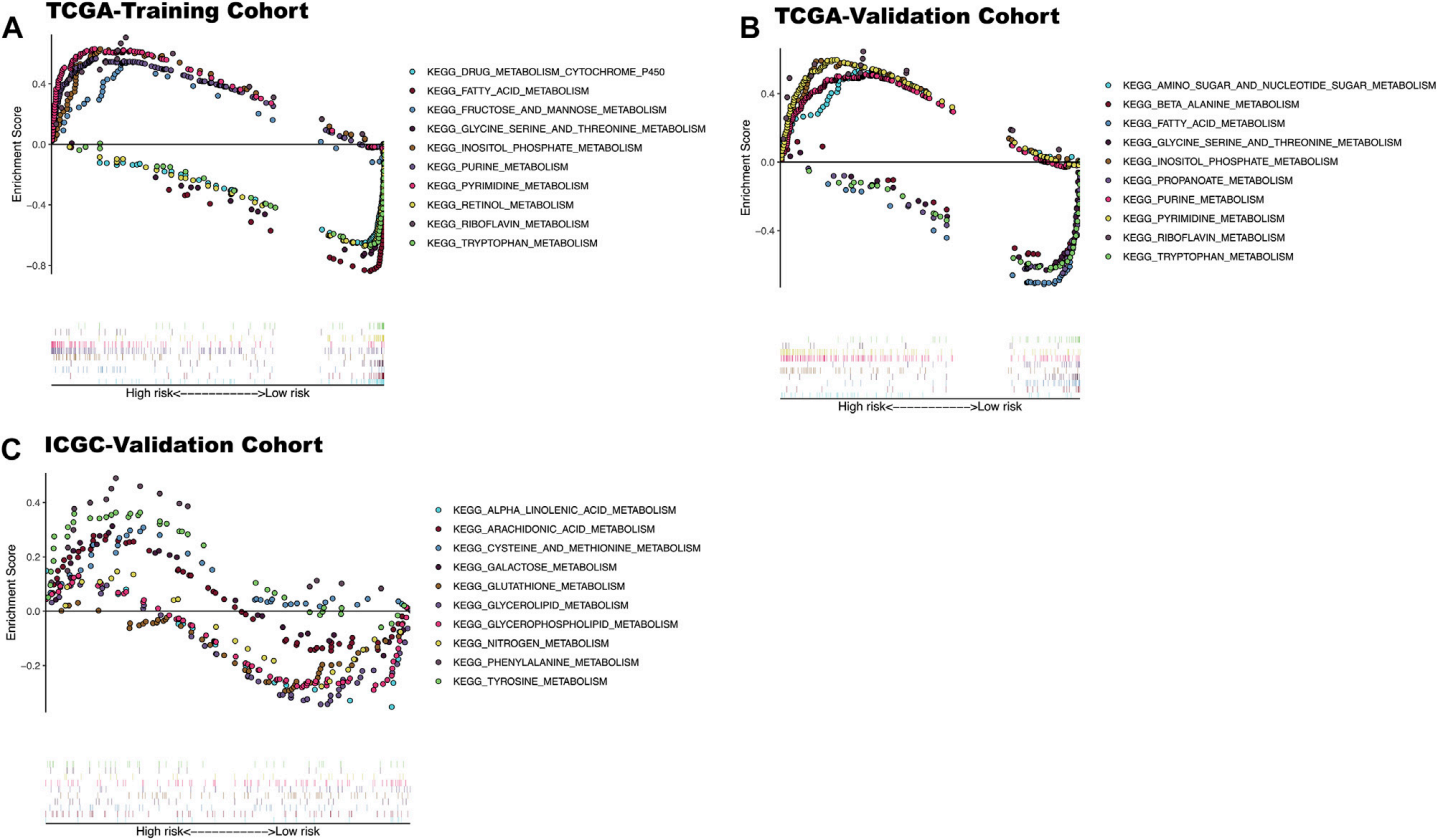
KEGG enrichment analysis results of three datasets. **(A)** The top five significantly enriched metabolic KEGG pathways in high and low-risk patients in TCGA-training cohort. **(B)** The top five significantly enriched metabolic KEGG pathways in high and low-risk patients In TCGA-Validation cohort. **(C)** The top five significantly enriched metabolic KEGG pathways in high and low risk patients In ICGC-validation cohort.

The enrichment analysis results included many metabolic-related signaling pathways. We selected the 5 most significantly related signaling pathways in the high and low-risk groups for display in Figure 7, whereas the relevant enrichment scores are shown in Table 2–4. The KEGG_PYRIMIDINE_METABOLISM and KEGG_PURINE_METABOLISM were two abundant metabolic pathways in high-risk scoring. The most significant enrichment in the low-risk grouping was the KEGG_FATTY_ACID_METABOLISM signal pathway.

**TABLE 2:** Top 5 metabolic signaling pathways significantly enriched in high/low risk groups of the training cohort.

No	Signaling pathways	Risk	ES	NES	Nom *p*-val	FDR *q*-val
1	KEGG_PYRIMIDINE_METABOLISM	High	0.63	1.99	0.000	0.004
2	KEGG_PURINE_METABOLISM	High	0.57	1.97	0.000	0.003
3	KEGG_RIBOFLAVIN_METABOLISM	High	0.71	1.83	0.002	0.012
4	KEGG_INOSITOL_PHOSPHATE_METABOLISM	High	0.63	1.77	0.004	0.019
5	KEGG_FRUCTOSE_AND_MANNOSE_METABOLISM	High	0.54	1.74	0.010	0.022
6	KEGG_FATTY_ACID_METABOLISM	Low	−0.85	−2.03	0.000	0.002
7	KEGG_RETINOL_METABOLISM	Low	−0.68	−1.96	0.000	0.005
8	KEGG_GLYCINE_SERINE_AND_THREONINE_METABOLISM	Low	−0.75	−1.93	0.004	0.005
9	KEGG_TRYPTOPHAN_METABOLISM	Low	−0.69	−1.92	0.002	0.004
10	KEGG_DRUG_METABOLISM_CYTOCHROME_P450	Low	−0.66	−1.90	0.000	0.005

Abbreviations: ES, enrichment score; NES, normalized enrichment score; Nom *p*-val, nominal *p*-value; FDR q-val, false discovery rate q-value.

**TABLE 3:** Top 5 metabolic signaling pathways significantly enriched in high/low risk groups of the TCGA-Validation cohort.

No	Signaling pathways	Risk	ES	NES	Nom *p-*val	FDR *q-*val
1	KEGG_PYRIMIDINE_METABOLISM	High	0.60	1.69	0.000	0.308
2	KEGG_PURINE_METABOLISM	High	0.51	1.53	0.007	0.226
3	KEGG_RIBOFLAVIN_METABOLISM	High	0.65	1.47	0.038	0.209
4	KEGG_INOSITOL_PHOSPHATE_METABOLISM	High	0.59	1.45	0.026	0.202
5	KEGG_AMINO_SUGAR_AND_NUCLEOTIDE_SUGAR_METABOLISM	High	0.53	1.41	0.044	0.230
6	KEGG_TRYPTOPHAN_METABOLISM	Low	−0.64	−1.65	0.004	0.080
7	KEGG_FATTY_ACID_METABOLISM	Low	−0.73	−1.64	0.020	0.064
8	KEGG_PROPANOATE_METABOLISM	Low	−0.68	−1.55	0.051	0.119
9	KEGG_BETA_ALANINE_METABOLISM	Low	−0.63	−1.53	0.048	0.124
10	KEGG_GLYCINE_SERINE_AND_THREONINE_METABOLISM	Low	−0.65	−1.48	0.060	0.145

Abbreviations: ES, enrichment score; NES, normalized enrichment score; Nom *p*-val, nominal *p*-value; FDR q-val, false discovery rate q-value.

**TABLE 4:** Top 5 metabolic signaling pathways significantly enriched in high/low risk groups of the ICGC-Validation cohort.

No	Signaling pathways	Risk	ES	NES	Nom *p-*val	FDR *q-*val
1	KEGG_PHENYLALANINE_METABOLISM	High	0.49	1.23	0.246	1
2	KEGG_CYSTEINE_AND_METHIONINE_METABOLISM	High	0.31	1.06	0.371	1
3	KEGG_TYROSINE_METABOLISM	High	0.36	1.02	0.440	1
4	KEGG_GALACTOSE_METABOLISM	High	0.31	1	0.461	1
5	KEGG_ARACHIDONIC_ACID_METABOLISM	High	0.28	0.95	0.541	1
6	KEGG_ALPHA_LINOLENIC_ACID_METABOLISM	Low	−0.44	−1.34	0.119	1
7	KEGG_GLYCEROLIPID_METABOLISM	Low	−0.36	−1.28	0.164	1
8	KEGG_GLYCEROPHOSPHOLIPID_METABOLISM	Low	−0.31	−1.16	0.276	1
9	KEGG_GLUTATHIONE_METABOLISM	Low	−0.30	−1.05	0.369	1
10	KEGG_NITROGEN_METABOLISM	Low	−0.31	−1.00	0.458	1

Abbreviations: ES, enrichment score; NES, normalized enrichment score; Nom *p*-val, nominal *p*-value; FDR q-val, false discovery rate q-value.

### Evaluation of Prognostic Model Efficacy Compared to ABIC, ALBI Score, ALBI Grade

**FIGURE 8:**
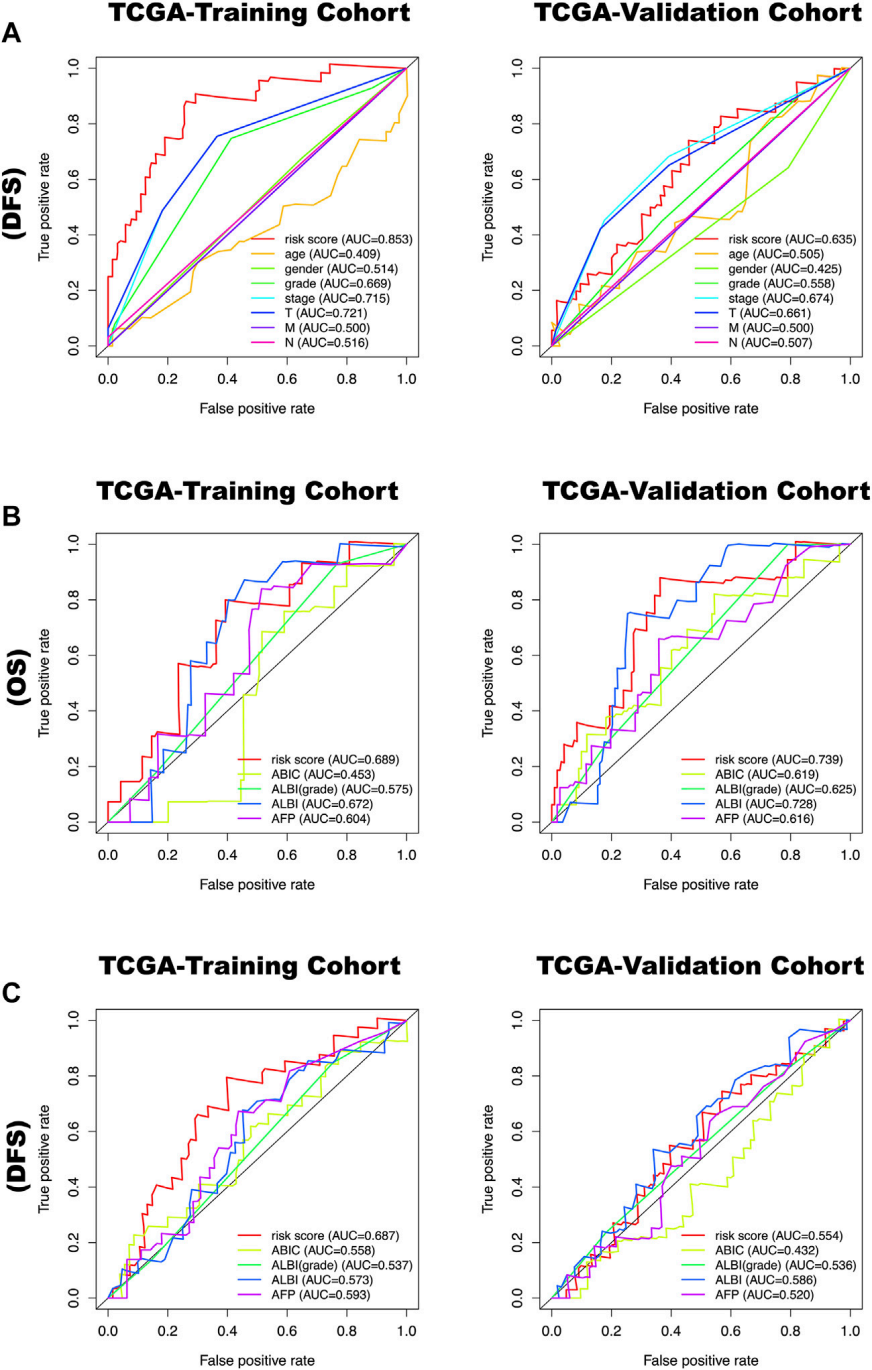
**(A)** The time-dependent ROC curves of risk score, age, gender, grade, stage, T, N, M for 1 year disease-free survival (DFS) in the TCGA-training cohort and TCGA-validation cohort. **(B)** The time-dependent ROC curves of risk score, ABIC, ALBI, ALBI grade, AFP for 1-year overall survival (OS) in the TCGA-training cohort and TCGA-validation cohort. **(C)** The time-dependent ROC curves of risk score, ABIC, ALBI, ALBI grade, AFP for 1-year disease-free survival (DFS) in the TCGA-training cohort and TCGA-validation cohort.

The time-dependent ROC curves of risk score, age, gender, grade, stage, T, N, M, ABIC, ALBI, ALBI grade, AFP for 1 year DFS and OS in the TCGA-training cohort and TCGA-validation cohort were determined. The results show that the predictive value of OS and DFS in the training cohort of the prognostic model based on the six metabolic genes is higher than for the above-mentioned metabolic predictors (Figure 8).

## Discussion

We constructed a gene signature composed of six metabolism-related mRNAs to investigate its role in HCC. We found six metabolism-related mRNAs in HCC through the LASSO regression model by analyzing the data from public datasets. This model performed well in predicting the prognosis of HCC patients, especially for OS within 3 and 5 years. Two articles were published that focused on the immune-based prognosis model of liver cancer (Wang et al., 2020; Zhang et al., 2020). The prognostic value of the metabolism-based prognostic model that we constructed (3-years AUC = 0.849) was higher than that of the immune-based prognostic model (3-years ROC = 0.711; 3-years ROC = 0.79), indicating that the six metabolic mRNAs signature that we constructed had a robust high prognostic value.

A recent study identified a four-gene metabolic signature and the signature that could efficiently stratify patients’ OS(Liu et al., 2020). The efficacy of our model was better than of the previous model, based on the following arguments. First, the prognostic value of our prognostic model in TCGA-training cohort (3-years AUC = 0.849) was higher than Liu’s (3-years ROC = 0.71), as was true for the TCGA-validation cohort (AUC = 0.757 vs AUC = 0.70). Second, we first tested the efficacy of our model internally in the TCGA data and then externally verified it in the Japanese cohort in the ICGC database. As an internationally cooperative tumor database, the ICGC database includes HCC cancer data that is more reliable and comprehensive. The Japanese cohort we used for external verification included 243 liver cancer cases, which was higher than Liu's 215 cases in GSE14520. What’s more, it has test power in the Japanese cohort, which shows that our model is also applicable to Asian races, and the external verification data used by Liu is the same as the TCGA data from the United States. Third, we did a differential expression analysis of the six genes used to construct the model in seven external HCC data (a total of 1,243 cases, tumor vs. normal = 720 vs. 523), and the results showed that the six genes were all highly expressed in liver cancer. Liu's research did not test the expression level of genes used to construct the model in other databases. Fourth, Liu’s model did not provide a relationship between the proposed model and DFS.

AFP is currently the most commonly used metabolic biomarker in clinical practice (Notarpaolo et al., 2017; Pinyol et al., 2019). It is often used to predict the prognosis of patients. We added AFP to compare it with our prediction model. The results suggested that our predictive model had a higher predictive value than AFP for both OS and DFS, whether it is in the training or validation cohort. Furthermore, we used same method to evaluate the efficacy of the prognostic model compared to Age-Bilirubin-International Normalized Ratio-Creatinine (ABIC) (Mitchell et al., 2017), the albumin-bilirubin (ALBI) score and ALBI grade (Mitchell et al., 2017). The results showed that the predictive value of OS and DFS in the training cohort of the prognostic model based on the six metabolic genes is higher than that of the above-mentioned metabolic predictors. Our nomogram has increased the testing power of clinical information, making its predictive power more accurate.

The advantage of the LASSO regression model is that it offers the possibility to delete genes that are highly correlated in order to reduce errors. Therefore, highly relevant mRNAs such as METTL6, POLR3A, POLR3G, AACS, etc. were not included when we constructed our model. The pathological grade and TNM stage are commonly used to predict the prognosis of HCC patients. In addition to the grade indicator, risk score, and TNM staging, these results were consistent with the training queue. The deviation of grade results had little effect on the model. In survival analysis, only TXNRD1 was not statistically significant, although the *p*-value was close to 0.05. When we reviewed the original data, we found that two of the samples with high expression of TXNRD1 had extremely long survival times, and the remaining patients had generally low survival times. The Kaplan-Meier (KM) graph also showed that the overall survival rate of patients with a high expression of TXNRD1 was lower than those with a low expression. This could be due to a sampling error, but when TXNRD1 and five other genes were used to form a model, it was statistically significant in predicting the survival rate, and its *p*-value was less than 0.001. In terms of gene expression verification, the size of the sample included in the differential analysis was large enough with high credibility. The prognostic model in our study has a high predictive accuracy with an AUC as high as 0.849. In addition, it showed a good performance in the verification set of two different databases, reflecting the stability of the model.

In the results of the enrichment analysis, we showed the top 5 significantly enriched metabolism-related signaling pathways. The KEGG_PYRIMIDINE_METABOLISM was the most abundant metabolic pathway in high risk scoring. Pyrimidine metabolism was very important in the process of cell proliferation. In HCC, the activation of the PYRIMIDINE signaling pathway could promote liver enlargement and tumor cell proliferation (Cox et al., 2016). The most significant enrichment in the low-risk grouping was the KEGG_FATTY_ACID_METABOLISM signal pathway. Lipid metabolism is one of the main sources of energy for tumor cells, which is very helpful for the adaptation of tumor cells to the microenvironment (Sangineto et al., 2020). The rest of the signaling pathways such as KEGG GLYCINE SERINE AND THREONINE METABOLISM (Li et al., 2015), KEGG_FRUCTOSE_AND_MANNOSE_METABOLISM (Huang et al., 2019b) and KEGG_DRUG_METABOLISM_CYTOCHROME_P450 (Nekvindova et al., 2020) have been reported in literature to be related to the malignant phenotype of HCC. These studies reflect that the six mRNAs in our model are closely related to HCC metabolism.

PRIM1 hasn’t been reported in liver cancer (Xu et al., 2016). However, in breast cancer and lung cancer it was found that the expression level of PRIM1 in tumor tissue was higher (Lee et al., 2019b). We think it is necessary to carry out more in-depth mechanism research to explore its mode of action in liver cancer. Like PRIM1, GMPS was found to be expressed higher in tumor tissues and peripheral blood of liver cancer patients (Zhang et al., 2017; Yin et al., 2019). UCK2 has been reported to be highly expressed in HCC tumors It promotes tumor cell metastasis and invasion through the Stat3 pathway (Zhou et al., 2018) and can be used as an independent prognostic factor (Huang et al., 2019a). TXNRD1 works as an oncogene in HCC and an TXNRD1 inhibitor significantly inhibits tumor growth (Fu et al., 2017; Lee et al., 2019a). TXNRD1 is an antioxidant enzyme regulated by the Nrf2/Keap1 pathway and it’s expression level is also negatively regulated by the non-coding RNA miR_125b_5p (Tuo et al., 2018; Hua et al., 2019). Tuo et al. found that TXNRD1 inhibitors can enhance the sensitivity of HCC tumor cells to sorafenib. In the future, an inhibitor suitable for clinical use could be developed for TXNRD1 and its combined use with sorafenib might improve the therapeutic effect. SMS, a polyamine biosynthetic enzyme, is overexpressed in patients with colorectal cancer and collaborates with MYC to promote colorectal cancer cell survival (Guo et al., 2020). However, little is known about SMS in liver disease and HCC and more experiments are necessary to reveal its role in HCC. Selenophosphate synthetase 2 (SEPHS 2) is essential for survival of cancer (Carlisle et al., 2020), but little research has focused on the role of SEPHS 1 in cancer and more functional experiments are needed.

Our study has some limitations. First, all of the data in our research come from databases, and the mRNA expression level and corresponding protein expression of the six genes for construction of the model need further clinical specimen verification. In addition, although our model can predict the prognosis of patients, there are many factors that affect the prognosis of HCC patients, such as tumor immunity, non-coding RNA, etc. Although our model has been well validated in the two large databases—TCGA and ICGC—we still need more data to fully verify the model’s reliability. Further experiments are required to verify the relationship between the six genes used to construct the model and the metabolism-related signaling pathways we obtained through enrichment analysis and their mechanism in the progression of HCC. What’s more, due to missing data for tumor number, BCLC staging and treatment type in TCGA, we can’t evaluate the efficacy of the prognostic model compared to the above-mentioned three variables.

In conclusion, we established a gene signature composed of six metabolism-related mRNAs that has significant value in predicting HCC survival and can be used to guide the adjuvant therapy for HCC.

## Data Availability

The original contributions presented in the study are included in the article/Supplementary Material, further inquiries can be directed to the corresponding author/s. Global list :Gene Expression Omnibus, https://portal.gdc.cancer.gov/, https://www.ncbi.nlm.nih.gov/, https://www.ncbi.nlm.nih.gov/geo/, https://www.proteinatlas.org/, human protein atlas, NCBI, ncbi.nlm.nih.gov/, ncbi.nlm.nih.gov/geo/, portal.gdc.cancer.gov/, proteinatlas.org/, The Cancer Genome Atlas
