# Supplementary material for: Six Metabolism Related mRNAs Predict the Prognosis of Patients With Hepatocellular Carcinoma
Source: Front Mol Biosci. 2021 Feb 25;8:621232. doi: 10.3389/fmolb.2021.621232 (PMC8045485; doi:10.3389/fmolb.2021.621232)
Supplement: Supplementary file 1 [file datasheet1.zip › Supplementary tables/Supplementary table 1.docx]

Supplementary table 1: The log fold change (logFC) and expression level of each differentially expressed genes (DEGS).

| gene | conMean | TreatMean | logFC | P Value | fdr-q-val |
| --- | --- | --- | --- | --- | --- |
| CYP4F2 | 60.3235252 | 29.46836538 | -1.0335537 | 6.21E-15 | 2.07E-14 |
| PTDSS2 | 1.662328 | 4.164425941 | 1.324912563 | 2.15E-23 | 2.63E-22 |
| GBA | 5.56522966 | 22.70087955 | 2.028235066 | 3.64E-28 | 4.21E-26 |
| AZIN2 | 0.124387656 | 0.277501557 | 1.157652541 | 9.96E-10 | 2.08E-09 |
| GSTZ1 | 11.81957816 | 4.287539533 | -1.46295667 | 2.84E-21 | 2.13E-20 |
| PDE7B | 0.930007826 | 0.456690251 | -1.02602686 | 1.31E-15 | 4.53E-15 |
| ADK | 19.8979868 | 13.99084463 | -0.50813941 | 1.05E-11 | 2.67E-11 |
| NPR2 | 2.597858634 | 5.96065675 | 1.19814837 | 0.000471399 | 0.000628015 |
| PLCE1 | 0.212974455 | 0.608776786 | 1.515232948 | 9.85E-19 | 5.02E-18 |
| INPP5J | 0.03995144 | 0.299135465 | 2.904479538 | 2.70E-11 | 6.56E-11 |
| SHMT1 | 52.7147148 | 32.16605069 | -0.71266692 | 1.45E-12 | 3.86E-12 |
| LPL | 0.141580397 | 1.102972743 | 2.961703712 | 1.86E-21 | 1.46E-20 |
| TAT | 265.3094254 | 154.2425759 | -0.78247488 | 2.12E-09 | 4.31E-09 |
| GRHPR | 62.909347 | 40.6577625 | -0.62974356 | 9.98E-12 | 2.55E-11 |
| POLR2I | 11.61097278 | 19.34012161 | 0.736108019 | 2.47E-10 | 5.51E-10 |
| PFKM | 0.866943006 | 2.25856606 | 1.381398054 | 3.07E-06 | 4.99E-06 |
| CYP2A6 | 536.9454174 | 278.4392503 | -0.94741284 | 2.29E-11 | 5.60E-11 |
| LPCAT2 | 0.474627134 | 1.255891704 | 1.40384558 | 0.000234735 | 0.000321719 |
| APRT | 28.6023808 | 54.41106794 | 0.927764906 | 3.86E-14 | 1.20E-13 |
| CYP2J2 | 34.1905172 | 23.20978966 | -0.5588628 | 5.17E-11 | 1.23E-10 |
| GSTT2 | 0.036948411 | 0.098586514 | 1.415878001 | 0.003668577 | 0.004592809 |
| CNDP1 | 6.560749296 | 1.024432657 | -2.67903544 | 1.45E-22 | 1.45E-21 |
| DPYS | 89.3997666 | 56.69502639 | -0.65704889 | 2.46E-09 | 4.96E-09 |
| APIP | 1.640616216 | 3.661128344 | 1.158050556 | 3.30E-22 | 3.07E-21 |
| PSAT1 | 58.4287532 | 37.24670641 | -0.64956565 | 3.01E-10 | 6.63E-10 |
| TDO2 | 85.2300468 | 35.60804767 | -1.25915879 | 2.05E-15 | 6.96E-15 |
| GYS2 | 38.5893622 | 12.55410139 | -1.62004443 | 4.34E-21 | 3.11E-20 |
| POLD3 | 0.842582454 | 2.064203063 | 1.292695122 | 2.33E-19 | 1.28E-18 |
| PISD | 2.83145052 | 4.389637725 | 0.632560561 | 7.78E-10 | 1.64E-09 |
| PLA2G4B | 0.017434527 | 0.047254432 | 1.438502371 | 4.96E-08 | 9.00E-08 |
| PYGB | 4.35291446 | 18.82163409 | 2.112338313 | 8.70E-26 | 2.52E-24 |
| AGPAT1 | 8.15078736 | 20.35100554 | 1.320088745 | 2.02E-24 | 3.34E-23 |
| NMNAT2 | 0.01628633 | 0.154646695 | 3.24724254 | 4.48E-07 | 7.74E-07 |
| LCT | 0.001303953 | 0.008682615 | 2.73523776 | 0.002015599 | 0.002571078 |
| PTGES | 0.297396069 | 3.200945654 | 3.428040699 | 0.014797686 | 0.017447054 |
| HMGCL | 40.4827466 | 24.59046419 | -0.71920821 | 2.52E-16 | 9.38E-16 |
| MARS | 4.17682288 | 10.38718963 | 1.314327497 | 1.39E-25 | 3.41E-24 |
| MTR | 1.27380779 | 3.363203 | 1.400688261 | 2.77E-16 | 1.02E-15 |
| CKM | 0.019812948 | 0.098969437 | 2.320539487 | 1.98E-05 | 3.01E-05 |
| GMPPA | 5.56356326 | 11.56588251 | 1.055794274 | 1.45E-24 | 2.61E-23 |
| CYP4A11 | 233.0618168 | 72.59752175 | -1.68272046 | 1.82E-24 | 3.12E-23 |
| NAA80 | 1.86636393 | 4.187413568 | 1.165829082 | 1.23E-18 | 6.06E-18 |
| CHKB | 0.422492212 | 1.182076973 | 1.484327329 | 9.12E-19 | 4.68E-18 |
| HAGHL | 0.048049798 | 0.557557535 | 3.536518411 | 1.02E-21 | 8.75E-21 |
| PIP4K2B | 3.30158812 | 7.811014925 | 1.242349865 | 1.56E-23 | 2.00E-22 |
| NMNAT3 | 0.695570616 | 1.378986438 | 0.987339375 | 1.16E-09 | 2.40E-09 |
| B4GALT6 | 0.297784155 | 0.661726029 | 1.151967038 | 2.51E-07 | 4.40E-07 |
| GMDS | 3.66597734 | 7.7145025 | 1.073375254 | 3.91E-10 | 8.47E-10 |
| HMGCS2 | 664.916598 | 435.8643833 | -0.60929407 | 5.83E-10 | 1.25E-09 |
| AMDHD2 | 2.031742526 | 4.730317108 | 1.219219314 | 1.28E-20 | 8.23E-20 |
| MARS2 | 1.96582365 | 2.821305799 | 0.52122914 | 4.85E-05 | 7.12E-05 |
| PLA2G4E | 0.004765986 | 0.0204447 | 2.10088031 | 7.67E-06 | 1.21E-05 |
| ODC1 | 18.19301946 | 33.29143499 | 0.871766053 | 1.65E-08 | 3.10E-08 |
| GALK2 | 1.588663302 | 2.448799751 | 0.624261408 | 4.17E-08 | 7.64E-08 |
| ADH4 | 804.750736 | 228.0260374 | -1.81934342 | 8.53E-21 | 5.66E-20 |
| AGPAT4 | 0.136465682 | 0.57271225 | 2.069272273 | 9.51E-10 | 1.99E-09 |
| SEPHS2 | 127.9557976 | 190.1067404 | 0.571164168 | 2.59E-10 | 5.75E-10 |
| NME3 | 9.19139972 | 22.42554503 | 1.286786564 | 6.38E-22 | 5.74E-21 |
| NAT1 | 2.219190636 | 1.399346689 | -0.66528017 | 2.09E-11 | 5.13E-11 |
| GPX2 | 106.9847132 | 267.7036539 | 1.323232162 | 0.010097916 | 0.012099574 |
| SGMS2 | 3.765996564 | 1.802598975 | -1.06295321 | 1.68E-05 | 2.57E-05 |
| B4GALT2 | 5.90955282 | 11.4905014 | 0.959320883 | 1.47E-17 | 6.36E-17 |
| PLA2G6 | 0.58048134 | 2.296062017 | 1.983840014 | 5.46E-24 | 7.75E-23 |
| HAO1 | 172.769988 | 92.22580563 | -0.90561024 | 3.35E-17 | 1.34E-16 |
| PDE1C | 0.008417989 | 0.083176516 | 3.304628652 | 2.04E-17 | 8.56E-17 |
| ABAT | 53.955323 | 26.73360559 | -1.01311087 | 3.57E-16 | 1.30E-15 |
| INPP4B | 0.149343996 | 0.294366749 | 0.97897548 | 0.001456753 | 0.001872968 |
| PIP4K2C | 4.43018188 | 8.973248829 | 1.018264488 | 9.69E-18 | 4.29E-17 |
| SPHK1 | 0.741709572 | 5.347719945 | 2.849997622 | 8.75E-05 | 0.000125878 |
| POLA2 | 0.664501946 | 2.587714342 | 1.961333037 | 8.15E-26 | 2.45E-24 |
| IMPA1 | 4.43702154 | 7.935114115 | 0.838659414 | 2.13E-12 | 5.61E-12 |
| LCLAT1 | 1.079431836 | 1.825327458 | 0.757883159 | 4.13E-11 | 9.85E-11 |
| CA5B | 0.142795734 | 0.550149828 | 1.945871693 | 6.48E-16 | 2.29E-15 |
| POLR2C | 9.07856552 | 14.29939552 | 0.655417896 | 2.19E-10 | 4.93E-10 |
| TSTA3 | 11.35656626 | 26.20332018 | 1.206222933 | 9.59E-18 | 4.27E-17 |
| SRM | 12.1914571 | 32.5068222 | 1.414871963 | 2.34E-23 | 2.83E-22 |
| UGT2B11 | 0.932454448 | 13.38406332 | 3.843339116 | 1.32E-12 | 3.57E-12 |
| HK3 | 1.755092942 | 1.065993519 | -0.71934876 | 1.59E-10 | 3.62E-10 |
| POLR2B | 4.42658506 | 7.625983009 | 0.784729173 | 7.24E-11 | 1.70E-10 |
| LPGAT1 | 8.05441224 | 21.31594928 | 1.404082087 | 2.17E-17 | 9.07E-17 |
| GLA | 3.24193056 | 11.36254672 | 1.809361132 | 1.20E-24 | 2.21E-23 |
| DNMT3A | 0.555289364 | 2.215351506 | 1.996223959 | 7.94E-24 | 1.09E-22 |
| POLR3F | 1.290860464 | 2.890671173 | 1.163071445 | 2.94E-25 | 6.80E-24 |
| DLAT | 3.66031604 | 7.052317534 | 0.946129215 | 1.16E-10 | 2.69E-10 |
| KMO | 8.11238004 | 3.338421404 | -1.28095916 | 8.62E-18 | 3.88E-17 |
| ADCY9 | 2.877553656 | 4.347904755 | 0.595477507 | 3.01E-06 | 4.91E-06 |
| ALDOB | 2772.70035 | 1401.228338 | -0.98459964 | 1.94E-14 | 6.20E-14 |
| CYP2C19 | 3.085339415 | 0.382142328 | -3.01324724 | 3.19E-17 | 1.29E-16 |
| PLA2G1B | 0.358601163 | 2.766570668 | 2.947646705 | 2.58E-09 | 5.17E-09 |
| AGXT | 394.753526 | 250.6042388 | -0.65554134 | 4.40E-10 | 9.48E-10 |
| ENTPD2 | 0.372271672 | 1.96584486 | 2.400721728 | 3.97E-12 | 1.02E-11 |
| DNMT1 | 1.168587338 | 4.380206253 | 1.906233242 | 7.50E-21 | 5.15E-20 |
| MGST3 | 5.56648038 | 10.14191067 | 0.865492151 | 1.91E-17 | 8.16E-17 |
| FPGT | 1.601297378 | 2.672968881 | 0.739201785 | 9.43E-13 | 2.59E-12 |
| CYP1A2 | 162.0610482 | 24.79812246 | -2.70823458 | 4.83E-24 | 6.99E-23 |
| CYP2C8 | 449.461848 | 100.5747926 | -2.1599299 | 6.04E-26 | 1.96E-24 |
| ACSL4 | 7.321489016 | 46.1219159 | 2.65524345 | 3.57E-12 | 9.31E-12 |
| DEGS1 | 7.60895486 | 17.27093585 | 1.182576051 | 6.59E-18 | 3.03E-17 |
| RDH8 | 0.002212514 | 0.177940518 | 6.329564415 | 8.28E-07 | 1.40E-06 |
| UPRT | 1.800134228 | 2.776221087 | 0.625017977 | 1.73E-11 | 4.30E-11 |
| POLR2H | 5.75387058 | 12.47900765 | 1.116898537 | 1.04E-22 | 1.09E-21 |
| ADH1B | 527.994414 | 221.1341117 | -1.25560108 | 3.38E-17 | 1.35E-16 |
| AC139530.1 | 0.504626934 | 1.532106421 | 1.602227395 | 9.25E-21 | 6.09E-20 |
| AHCYL2 | 1.248491948 | 1.918117883 | 0.619504871 | 8.41E-07 | 1.42E-06 |
| PTGES2 | 7.21657504 | 16.7167287 | 1.211906346 | 8.10E-22 | 7.21E-21 |
| ADCY2 | 0.001111836 | 0.006195357 | 2.478242936 | 0.016679715 | 0.019580535 |
| LCMT2 | 0.797014992 | 1.12892053 | 0.502265164 | 4.15E-07 | 7.20E-07 |
| GLS | 1.201759816 | 4.164693323 | 1.793061676 | 3.87E-12 | 1.00E-11 |
| NT5C | 5.03591866 | 11.91640459 | 1.242622127 | 8.44E-19 | 4.36E-18 |
| LPCAT4 | 0.587157698 | 1.661035346 | 1.500262836 | 1.57E-10 | 3.58E-10 |
| OTC | 88.002804 | 58.70658403 | -0.58402718 | 6.74E-07 | 1.15E-06 |
| AMPD2 | 4.28833292 | 7.542024427 | 0.814534911 | 7.54E-13 | 2.09E-12 |
| RRM2B | 3.53135628 | 5.709601422 | 0.693167654 | 1.77E-08 | 3.31E-08 |
| ACHE | 0.686224915 | 1.858858895 | 1.437663848 | 0.032860592 | 0.037970156 |
| ASNS | 0.500573577 | 2.570302345 | 2.36028403 | 2.43E-09 | 4.91E-09 |
| HAAO | 80.8500472 | 45.99512303 | -0.81376772 | 3.64E-12 | 9.44E-12 |
| CA4 | 0.042076729 | 0.569382669 | 3.758304113 | 5.34E-07 | 9.17E-07 |
| ALDH1B1 | 67.6148248 | 40.63371028 | -0.73466249 | 5.75E-11 | 1.36E-10 |
| DMGDH | 31.7591144 | 15.47932348 | -1.03682826 | 5.46E-16 | 1.95E-15 |
| MTHFD2 | 0.687717227 | 1.248735294 | 0.860580299 | 0.006481558 | 0.00790672 |
| GGT5 | 7.32869966 | 4.290134866 | -0.77253424 | 1.15E-12 | 3.11E-12 |
| CYP2C9 | 287.583338 | 118.3820532 | -1.28052971 | 2.02E-17 | 8.51E-17 |
| GPD2 | 0.764990188 | 1.774788278 | 1.214133781 | 1.94E-12 | 5.11E-12 |
| CKMT1B | 0.007144548 | 0.294667545 | 5.366101652 | 0.004188776 | 0.005187934 |
| CEL | 0.033783259 | 0.531241933 | 3.974988605 | 3.70E-16 | 1.34E-15 |
| POLE2 | 0.325707436 | 1.639062106 | 2.331221958 | 5.74E-23 | 6.29E-22 |
| NEU3 | 0.572362622 | 1.294232685 | 1.177095651 | 1.28E-17 | 5.57E-17 |
| CKMT1A | 0.006887984 | 0.134522648 | 4.287623557 | 8.85E-07 | 1.49E-06 |
| GLS2 | 4.778706618 | 1.78680446 | -1.41923844 | 1.24E-15 | 4.32E-15 |
| PLPP1 | 14.99118618 | 30.87743991 | 1.042438601 | 9.08E-10 | 1.90E-09 |
| PRIM1 | 0.936742084 | 3.706683544 | 1.984405165 | 4.14E-21 | 2.99E-20 |
| PTGIS | 2.191950054 | 1.166763256 | -0.90970307 | 2.41E-14 | 7.66E-14 |
| ACSM2A | 35.2888359 | 22.45481559 | -0.65218697 | 3.27E-08 | 6.04E-08 |
| AKR1C3 | 17.68298576 | 83.41396337 | 2.237927015 | 1.18E-23 | 1.57E-22 |
| PHGDH | 23.51638218 | 13.43361449 | -0.8078186 | 2.77E-11 | 6.68E-11 |
| CKB | 3.60284462 | 23.67772586 | 2.716322184 | 3.20E-06 | 5.19E-06 |
| HK1 | 1.756529668 | 3.532845207 | 1.008102594 | 0.004998106 | 0.006143347 |
| PLA2G7 | 1.73344392 | 4.023914912 | 1.214958635 | 0.000584406 | 0.000773478 |
| ADH6 | 89.808434 | 46.97759442 | -0.9348781 | 6.33E-14 | 1.93E-13 |
| UCKL1 | 4.9052273 | 11.12656123 | 1.181615888 | 1.80E-23 | 2.28E-22 |
| PYCR3 | 2.93010406 | 9.072809425 | 1.630597454 | 9.42E-25 | 1.80E-23 |
| CHKA | 3.36662532 | 11.5937026 | 1.78396631 | 5.27E-22 | 4.85E-21 |
| UGDH | 22.73636822 | 41.03832833 | 0.85197014 | 0.000206002 | 0.000284746 |
| NME4 | 16.31808562 | 24.98593807 | 0.614644568 | 0.000109914 | 0.000155647 |
| RDH16 | 121.453585 | 43.35288581 | -1.48620514 | 1.45E-19 | 8.24E-19 |
| POLD2 | 19.6641202 | 29.83523623 | 0.60145156 | 7.12E-09 | 1.38E-08 |
| P4HA2 | 1.008157448 | 4.303512033 | 2.093793535 | 1.64E-22 | 1.60E-21 |
| HMGCS1 | 26.26538034 | 41.55101209 | 0.661721142 | 0.000113855 | 0.000160946 |
| DGKD | 0.77171494 | 1.905129995 | 1.303749501 | 4.07E-19 | 2.17E-18 |
| OPLAH | 8.82522278 | 16.89672561 | 0.937039094 | 2.09E-10 | 4.71E-10 |
| UCK1 | 8.54689772 | 14.03707748 | 0.715769834 | 3.83E-13 | 1.11E-12 |
| NANS | 4.36062666 | 7.892830021 | 0.856007202 | 7.18E-17 | 2.78E-16 |
| PCYT2 | 15.79421824 | 27.65662255 | 0.808228454 | 6.16E-10 | 1.31E-09 |
| NME2 | 6.86305604 | 19.23153189 | 1.486550645 | 1.71E-21 | 1.40E-20 |
| PDE2A | 2.178131344 | 1.126729193 | -0.95095015 | 1.15E-13 | 3.47E-13 |
| OGDHL | 26.5042672 | 14.50832449 | -0.86934374 | 4.39E-13 | 1.26E-12 |
| CDS2 | 2.7498919 | 4.632668795 | 0.752468636 | 1.26E-14 | 4.13E-14 |
| PGD | 20.44094368 | 39.89972749 | 0.964917092 | 3.83E-09 | 7.60E-09 |
| AMPD3 | 0.254917141 | 0.543059142 | 1.091080935 | 7.02E-05 | 0.000102034 |
| SMPD4 | 2.28095394 | 5.437838743 | 1.253396055 | 2.14E-22 | 2.06E-21 |
| SYNJ2 | 1.00822186 | 2.334073703 | 1.211036978 | 8.08E-10 | 1.70E-09 |
| GYS1 | 1.954834784 | 4.327244832 | 1.146402068 | 3.57E-18 | 1.70E-17 |
| CS | 5.37878536 | 13.8183489 | 1.361232919 | 1.45E-22 | 1.45E-21 |
| POLD4 | 8.05781742 | 12.54161247 | 0.638261825 | 5.34E-09 | 1.05E-08 |
| AKR1B10 | 21.56249119 | 348.4054764 | 4.014171528 | 1.73E-11 | 4.30E-11 |
| POLA1 | 0.530661838 | 1.544546194 | 1.541318312 | 4.30E-19 | 2.28E-18 |
| ACAA2 | 105.504459 | 52.75512735 | -0.99992075 | 5.19E-20 | 3.09E-19 |
| AOX1 | 221.5975442 | 121.9637437 | -0.86148955 | 1.55E-11 | 3.88E-11 |
| TYRP1 | 0.008600457 | 0.267724997 | 4.960194749 | 0.000375099 | 0.000506384 |
| FMO1 | 0.209436746 | 1.621167947 | 2.952447066 | 0.003981116 | 0.004953463 |
| PLPP3 | 35.3993676 | 17.43031475 | -1.02212497 | 3.08E-17 | 1.25E-16 |
| ASMT | 0.009882152 | 0.02670528 | 1.434227825 | 0.000871412 | 0.001143993 |
| CA12 | 0.325978387 | 3.7360177 | 3.518653069 | 1.48E-09 | 3.06E-09 |
| INMT | 9.65272184 | 2.507403196 | -1.9447418 | 7.33E-21 | 5.08E-20 |
| PRUNE1 | 3.4628476 | 9.611363235 | 1.472782175 | 5.02E-23 | 5.65E-22 |
| ACSL3 | 6.99338192 | 11.86570804 | 0.76273599 | 6.24E-09 | 1.21E-08 |
| PYCR1 | 0.861568492 | 8.121217599 | 3.236658648 | 9.35E-06 | 1.46E-05 |
| ACP1 | 11.20816652 | 18.54387434 | 0.7263924 | 8.05E-21 | 5.39E-20 |
| PHPT1 | 13.54757584 | 48.38776828 | 1.836607678 | 3.87E-26 | 1.53E-24 |
| AMDHD1 | 41.6883632 | 23.51702173 | -0.82593936 | 1.15E-12 | 3.11E-12 |
| CA8 | 0.013419862 | 0.117481387 | 3.129990477 | 5.78E-12 | 1.49E-11 |
| PIP5K1C | 1.467138022 | 3.719134168 | 1.341962194 | 3.88E-23 | 4.55E-22 |
| PDE6C | 0.038095006 | 0.11785532 | 1.629343082 | 2.78E-13 | 8.17E-13 |
| INPP4A | 0.504742884 | 0.934387362 | 0.888472096 | 7.54E-11 | 1.76E-10 |
| MAT1A | 492.371978 | 208.7499778 | -1.23797261 | 2.22E-21 | 1.73E-20 |
| MTHFD1 | 36.8332948 | 23.39997829 | -0.65450326 | 3.30E-11 | 7.89E-11 |
| ADCY3 | 0.561033466 | 1.082943581 | 0.948799348 | 0.000235869 | 0.000322726 |
| GSTM5 | 0.363642937 | 0.231850861 | -0.64932548 | 3.60E-10 | 7.85E-10 |
| NME6 | 1.050427416 | 2.501002343 | 1.251529933 | 3.81E-27 | 2.57E-25 |
| PKM | 4.01863632 | 25.24352982 | 2.65113573 | 1.86E-14 | 6.02E-14 |
| FMO3 | 137.9187216 | 93.88484103 | -0.55485417 | 3.50E-08 | 6.45E-08 |
| PI4KB | 4.0879725 | 11.41793196 | 1.481843974 | 1.67E-26 | 8.44E-25 |
| CANT1 | 4.13244666 | 10.66555898 | 1.367891476 | 6.59E-25 | 1.33E-23 |
| IMPDH2 | 12.91924434 | 36.52956009 | 1.499542692 | 3.89E-24 | 5.91E-23 |
| PLCD3 | 0.266926144 | 1.228215767 | 2.202051505 | 8.51E-16 | 2.98E-15 |
| MDH1 | 20.8540302 | 33.17323875 | 0.669693649 | 3.56E-14 | 1.11E-13 |
| TPI1 | 83.9320608 | 139.4822363 | 0.73278749 | 5.71E-14 | 1.75E-13 |
| SYNJ1 | 0.648474406 | 0.91861337 | 0.502408146 | 0.000217285 | 0.00029983 |
| ALAS1 | 159.56769 | 100.1414548 | -0.67212924 | 1.81E-09 | 3.71E-09 |
| UXS1 | 1.5551954 | 4.725752008 | 1.603448067 | 1.67E-26 | 8.44E-25 |
| ITPKB | 0.905496968 | 1.49022587 | 0.718749297 | 4.85E-05 | 7.12E-05 |
| GPD1L | 0.656858692 | 1.902400678 | 1.534166188 | 5.88E-09 | 1.14E-08 |
| GSTM2 | 0.271784875 | 0.548345501 | 1.012620017 | 0.000818116 | 0.001077519 |
| FBP2 | 0.038394142 | 0.087854528 | 1.194230413 | 0.002536832 | 0.003215703 |
| CBR3 | 0.238376351 | 1.432379474 | 2.587100731 | 8.94E-16 | 3.12E-15 |
| DCK | 1.436440984 | 3.648930365 | 1.344974898 | 1.32E-15 | 4.55E-15 |
| CYP3A43 | 2.620158394 | 1.416590761 | -0.88723099 | 1.35E-12 | 3.62E-12 |
| DNMT3L | 0.424289348 | 0.291648168 | -0.54081945 | 2.06E-05 | 3.10E-05 |
| LPCAT1 | 1.635662076 | 6.94731366 | 2.086580512 | 1.78E-16 | 6.71E-16 |
| GPAT4 | 5.23151944 | 9.836442457 | 0.910906608 | 1.77E-12 | 4.69E-12 |
| CYB5R3 | 30.237305 | 43.89137451 | 0.53760789 | 2.01E-07 | 3.56E-07 |
| ALOX15 | 0.021905219 | 0.096188973 | 2.134596898 | 8.16E-06 | 1.28E-05 |
| ARSA | 13.84392328 | 23.18197984 | 0.743750933 | 1.06E-09 | 2.20E-09 |
| PDE5A | 0.195980374 | 0.503871718 | 1.362347291 | 1.58E-06 | 2.62E-06 |
| CYP2E1 | 530.2645806 | 266.8712181 | -0.99056867 | 5.25E-11 | 1.24E-10 |
| AOC2 | 0.219990869 | 0.495573818 | 1.171656326 | 1.73E-09 | 3.55E-09 |
| EPHX1 | 524.41677 | 1035.59981 | 0.981680879 | 7.88E-06 | 1.24E-05 |
| MTHFD2L | 1.241255578 | 0.761231939 | -0.7053922 | 5.19E-10 | 1.11E-09 |
| PFKFB4 | 0.151297184 | 0.756509371 | 2.321972815 | 5.38E-18 | 2.50E-17 |
| ECHS1 | 468.98201 | 301.2351756 | -0.63864234 | 5.40E-13 | 1.52E-12 |
| DBH | 10.78850207 | 1.804720176 | -2.5796475 | 1.78E-24 | 3.12E-23 |
| ADH1A | 388.209718 | 164.8505374 | -1.23567764 | 5.65E-17 | 2.20E-16 |
| ATIC | 7.75802162 | 18.69461756 | 1.268862257 | 1.56E-23 | 2.00E-22 |
| FTCD | 155.5260496 | 86.5196214 | -0.84605699 | 6.71E-13 | 1.87E-12 |
| GSTO2 | 0.303990672 | 0.880769726 | 1.534737826 | 0.001201774 | 0.001557499 |
| GPAT2 | 0.090898479 | 0.478071167 | 2.394897342 | 2.26E-10 | 5.08E-10 |
| ARG2 | 0.49592443 | 2.010793536 | 2.019572755 | 7.62E-05 | 0.000110012 |
| GBA3 | 29.65604208 | 9.090840342 | -1.7058405 | 2.15E-20 | 1.33E-19 |
| ACSM1 | 3.973052599 | 15.02401635 | 1.91895074 | 0.000302397 | 0.000411667 |
| UGT1A1 | 27.79325188 | 16.52163987 | -0.75037775 | 8.92E-09 | 1.72E-08 |
| ENOPH1 | 5.50486998 | 11.72488047 | 1.090792821 | 2.78E-21 | 2.10E-20 |
| GPX1 | 124.116905 | 222.3074623 | 0.840856749 | 1.77E-10 | 4.01E-10 |
| CDIPT | 20.9301402 | 32.18929631 | 0.620999063 | 1.08E-11 | 2.74E-11 |
| AGPS | 3.7115833 | 5.295453451 | 0.51271948 | 2.80E-05 | 4.17E-05 |
| ACSM4 | 0.008317656 | 0.032206493 | 1.953102573 | 1.13E-06 | 1.90E-06 |
| IDO1 | 0.444306793 | 1.213901216 | 1.450022921 | 0.000360873 | 0.000487992 |
| PIK3C2B | 0.775023298 | 2.386662492 | 1.622682978 | 2.26E-18 | 1.10E-17 |
| NNMT | 469.3829578 | 140.666585 | -1.7384858 | 1.15E-17 | 5.06E-17 |
| HEXA | 3.18901968 | 6.698987527 | 1.070830065 | 1.23E-18 | 6.06E-18 |
| BHMT | 188.5596978 | 89.71365222 | -1.0716219 | 2.19E-13 | 6.51E-13 |
| ADH1C | 476.144326 | 233.435638 | -1.02837411 | 1.14E-12 | 3.10E-12 |
| CTPS2 | 1.630246474 | 3.255587356 | 0.997827751 | 8.08E-18 | 3.66E-17 |
| PGM1 | 59.3355864 | 38.43717944 | -0.62639514 | 1.11E-13 | 3.36E-13 |
| ACADSB | 72.1887034 | 32.77565001 | -1.1391487 | 1.46E-18 | 7.13E-18 |
| MBOAT7 | 4.30759108 | 10.22851875 | 1.247644029 | 2.35E-24 | 3.80E-23 |
| CBR1 | 58.780441 | 99.21114632 | 0.755166033 | 0.001746125 | 0.002237913 |
| ENTPD1 | 0.634465836 | 1.48168498 | 1.223624363 | 2.79E-19 | 1.51E-18 |
| PSPH | 1.983490216 | 9.025365702 | 2.185944109 | 3.94E-24 | 5.91E-23 |
| ACADM | 31.879362 | 20.11711322 | -0.66419946 | 2.07E-11 | 5.11E-11 |
| BLVRA | 3.276705816 | 12.07960422 | 1.882255129 | 5.93E-14 | 1.81E-13 |
| GSTM4 | 3.97687114 | 6.010324011 | 0.595808951 | 0.003231848 | 0.004071224 |
| GMPR2 | 7.85884262 | 12.66631626 | 0.688608242 | 1.09E-18 | 5.51E-18 |
| ASS1 | 584.625332 | 252.8417532 | -1.20927762 | 1.11E-18 | 5.59E-18 |
| PLCB1 | 0.225675628 | 1.091508615 | 2.274000988 | 4.77E-14 | 1.48E-13 |
| ADPRM | 2.7431114 | 3.986350382 | 0.53925531 | 1.35E-05 | 2.08E-05 |
| AOC1 | 1.798183299 | 0.745771443 | -1.26973463 | 0.001027935 | 0.001342947 |
| DGKH | 0.113162972 | 0.327426191 | 1.532767749 | 3.75E-16 | 1.36E-15 |
| PCK2 | 145.7806962 | 90.77617087 | -0.68341416 | 6.45E-11 | 1.52E-10 |
| GNPDA2 | 0.319322526 | 0.662430617 | 1.052755026 | 1.31E-10 | 3.01E-10 |
| HEMK1 | 0.65588727 | 1.173926415 | 0.8398222 | 7.25E-21 | 5.06E-20 |
| PAPSS1 | 2.28088236 | 5.466021302 | 1.260899043 | 7.56E-17 | 2.90E-16 |
| AKR1C2 | 16.24967136 | 50.10020952 | 1.624406096 | 5.01E-06 | 7.97E-06 |
| RDH10 | 9.06576346 | 15.41329115 | 0.765674524 | 1.50E-06 | 2.49E-06 |
| POLR2J3 | 0.081768923 | 0.192146356 | 1.232581075 | 3.81E-18 | 1.80E-17 |
| UCK2 | 0.97723134 | 4.97742167 | 2.348626575 | 2.02E-28 | 4.10E-26 |
| NAT2 | 25.52141146 | 4.833929648 | -2.40043974 | 1.20E-25 | 3.35E-24 |
| METTL2B | 1.507055692 | 2.85584579 | 0.922185347 | 1.48E-21 | 1.24E-20 |
| IMPDH1 | 1.350320208 | 4.233778779 | 1.648644327 | 7.85E-07 | 1.34E-06 |
| ACOT12 | 21.45356354 | 13.71772584 | -0.64517598 | 9.38E-09 | 1.80E-08 |
| PDHA1 | 13.28151162 | 19.02741134 | 0.518659943 | 7.54E-11 | 1.76E-10 |
| POLR1A | 0.958182464 | 2.696126111 | 1.492515664 | 3.87E-26 | 1.53E-24 |
| CYB5R1 | 6.89941468 | 17.42151749 | 1.336324415 | 1.52E-25 | 3.62E-24 |
| POLE4 | 7.38593728 | 10.66305657 | 0.52976813 | 0.000190569 | 0.000263865 |
| BUD23 | 4.074106 | 8.36906785 | 1.038583427 | 1.30E-25 | 3.41E-24 |
| DUT | 3.95256818 | 10.54330863 | 1.415465421 | 3.56E-24 | 5.55E-23 |
| SEPHS1 | 7.92983666 | 13.77461218 | 0.796648647 | 1.75E-20 | 1.09E-19 |
| GGCT | 7.3906693 | 16.97966798 | 1.200031323 | 1.92E-23 | 2.39E-22 |
| MLYCD | 2.48341314 | 1.547413766 | -0.68246527 | 8.55E-15 | 2.84E-14 |
| DNMT3B | 0.128279551 | 0.715787579 | 2.480240297 | 7.59E-21 | 5.15E-20 |
| POLR2K | 10.2636964 | 29.85350878 | 1.540350105 | 2.97E-28 | 4.21E-26 |
| LYPLA1 | 8.63802894 | 13.72384588 | 0.667910775 | 2.84E-09 | 5.67E-09 |
| GCDH | 24.69117048 | 14.79007361 | -0.739366 | 2.95E-12 | 7.71E-12 |
| ACSL6 | 0.2617771 | 1.020156731 | 1.962380012 | 0.035460273 | 0.04074159 |
| POLR3C | 2.99792546 | 7.304838 | 1.284887766 | 1.45E-22 | 1.45E-21 |
| GPI | 20.7880496 | 42.19970111 | 1.021478374 | 1.93E-16 | 7.25E-16 |
| GPX4 | 118.53575 | 188.6938862 | 0.670725443 | 1.28E-10 | 2.95E-10 |
| CAT | 172.3659592 | 98.46953686 | -0.8077255 | 2.28E-14 | 7.27E-14 |
| PGS1 | 1.087267554 | 2.837714227 | 1.384022308 | 3.10E-24 | 4.92E-23 |
| PTDSS1 | 6.74127936 | 12.91530242 | 0.937987108 | 5.06E-15 | 1.70E-14 |
| ACADL | 4.060608256 | 2.250819133 | -0.85124572 | 6.55E-12 | 1.68E-11 |
| DGKA | 0.329520905 | 0.633643682 | 0.943301801 | 2.37E-05 | 3.57E-05 |
| POLR2F | 0.004665332 | 0.009967736 | 1.095286074 | 0.000318484 | 0.000432114 |
| ME3 | 0.531760212 | 1.606608404 | 1.595170589 | 3.70E-15 | 1.25E-14 |
| PRPS1 | 12.66083042 | 18.44318764 | 0.542715993 | 3.69E-06 | 5.95E-06 |
| ALDH6A1 | 64.384127 | 28.49727636 | -1.17588102 | 9.37E-20 | 5.46E-19 |
| DGAT1 | 11.44228732 | 19.98911916 | 0.804839423 | 2.18E-09 | 4.43E-09 |
| ALDOA | 20.55360858 | 72.12748631 | 1.811157437 | 2.08E-19 | 1.16E-18 |
| CPT2 | 25.1462894 | 16.46242324 | -0.61116882 | 2.48E-12 | 6.49E-12 |
| AGK | 1.495999866 | 2.767032531 | 0.887229559 | 4.65E-16 | 1.67E-15 |
| GNPDA1 | 2.73301806 | 7.292774088 | 1.41597271 | 3.86E-20 | 2.33E-19 |
| CES5A | 1.156581576 | 0.643541115 | -0.8457628 | 2.67E-09 | 5.34E-09 |
| AADAT | 11.0372536 | 2.431650612 | -2.18237337 | 7.44E-26 | 2.32E-24 |
| NUDT5 | 9.44814408 | 17.61621736 | 0.898801304 | 1.61E-21 | 1.33E-20 |
| GLUL | 58.1799336 | 366.9451525 | 2.656970884 | 0.000190569 | 0.000263865 |
| PRIM2 | 0.502543684 | 1.859031108 | 1.887229998 | 4.42E-27 | 2.75E-25 |
| TH | 0.103477478 | 0.041963227 | -1.30211928 | 7.05E-23 | 7.61E-22 |
| RRM1 | 3.92014216 | 10.15498263 | 1.373209893 | 1.84E-21 | 1.46E-20 |
| CHST12 | 0.378759634 | 0.713920394 | 0.91448063 | 2.32E-13 | 6.85E-13 |
| UGT2B7 | 279.779916 | 115.0979264 | -1.28143056 | 6.96E-18 | 3.18E-17 |
| DGUOK | 11.27155494 | 19.37659762 | 0.781628714 | 1.50E-13 | 4.49E-13 |
| WARS2 | 1.72666976 | 3.099357597 | 0.843977038 | 2.58E-16 | 9.53E-16 |
| CYP1B1 | 1.337915968 | 5.94969667 | 2.152828612 | 0.006928705 | 0.008426803 |
| RPE65 | 0.00181145 | 0.009259958 | 2.353860307 | 1.08E-06 | 1.81E-06 |
| DTYMK | 2.81252568 | 11.25842326 | 2.001066619 | 4.78E-28 | 4.84E-26 |
| GAA | 20.41744244 | 34.96541419 | 0.776126435 | 1.55E-11 | 3.88E-11 |
| ADSS | 4.6381151 | 9.471381832 | 1.030036301 | 2.47E-17 | 1.01E-16 |
| IL4I1 | 0.408676109 | 1.900420927 | 2.217289189 | 4.63E-08 | 8.44E-08 |
| PFKP | 0.971925658 | 4.860376484 | 2.322150197 | 1.35E-06 | 2.26E-06 |
| TXNRD1 | 6.76314148 | 28.10662419 | 2.055144747 | 1.93E-17 | 8.20E-17 |
| SMPD3 | 0.70683542 | 0.332301761 | -1.0888804 | 4.31E-17 | 1.70E-16 |
| CHST13 | 13.29861354 | 25.15093577 | 0.919336234 | 5.40E-07 | 9.24E-07 |
| RRM2 | 0.469398657 | 6.378298565 | 3.764286013 | 3.18E-26 | 1.43E-24 |
| CPT1C | 0.112497877 | 0.356503529 | 1.664018582 | 1.18E-13 | 3.56E-13 |
| UGP2 | 58.5313736 | 40.07029916 | -0.54667685 | 1.31E-10 | 3.01E-10 |
| PPAT | 0.863819914 | 1.765653998 | 1.031400175 | 3.59E-13 | 1.05E-12 |
| GLO1 | 32.9487238 | 51.30883037 | 0.638984552 | 5.19E-09 | 1.02E-08 |
| LCAT | 68.1154072 | 16.82335661 | -2.01751558 | 2.17E-26 | 1.03E-24 |
| CYP26A1 | 6.569500214 | 0.658808858 | -3.31785176 | 1.48E-21 | 1.24E-20 |
| NME1-NME2 | 0.318942837 | 0.531576005 | 0.736978107 | 0.000431791 | 0.000579057 |
| ADCY6 | 1.052476086 | 3.845742664 | 1.869474775 | 3.47E-25 | 7.81E-24 |
| MTMR2 | 0.825414542 | 1.902368228 | 1.204605765 | 2.99E-11 | 7.16E-11 |
| LTA4H | 4.31542466 | 7.329835003 | 0.764278188 | 1.35E-18 | 6.65E-18 |
| POLR3GL | 12.67931058 | 19.32978751 | 0.608349475 | 1.82E-09 | 3.71E-09 |
| POLR3H | 2.7771828 | 4.797098075 | 0.788539794 | 1.59E-16 | 6.02E-16 |
| ACP6 | 0.71604374 | 1.761317951 | 1.298535743 | 2.41E-20 | 1.47E-19 |
| PPOX | 1.196744276 | 4.192724463 | 1.808773117 | 1.44E-29 | 7.49E-27 |
| ACSL1 | 181.0926394 | 77.88915995 | -1.21723344 | 8.80E-18 | 3.94E-17 |
| PEMT | 52.8566338 | 30.87396532 | -0.77569376 | 4.96E-14 | 1.53E-13 |
| PLCD4 | 0.125425913 | 0.438557777 | 1.805931481 | 1.23E-18 | 6.06E-18 |
| NOS2 | 0.097262382 | 0.402505335 | 2.049054083 | 5.21E-13 | 1.48E-12 |
| FHIT | 0.791113118 | 2.26275842 | 1.516126666 | 6.30E-22 | 5.74E-21 |
| HMOX1 | 45.83893488 | 27.73183896 | -0.72503023 | 9.52E-09 | 1.82E-08 |
| TK1 | 2.468539172 | 20.42635341 | 3.048702228 | 5.30E-26 | 1.87E-24 |
| PLCG1 | 1.260780372 | 3.995510695 | 1.664062936 | 1.77E-21 | 1.44E-20 |
| TPH1 | 0.011207079 | 0.069008447 | 2.622362652 | 6.41E-10 | 1.36E-09 |
| GMPPB | 1.87040842 | 4.208787714 | 1.170051414 | 8.29E-23 | 8.72E-22 |
| ACACB | 8.46489348 | 5.654933634 | -0.58198182 | 3.48E-09 | 6.92E-09 |
| ASPA | 1.222286356 | 0.511516308 | -1.25673018 | 1.83E-17 | 7.86E-17 |
| NAMPT | 30.29449552 | 16.41230958 | -0.88427741 | 2.28E-07 | 4.01E-07 |
| SPTLC1 | 3.060500882 | 4.809781218 | 0.652203488 | 1.51E-05 | 2.31E-05 |
| POLR2J | 16.07014268 | 32.76108605 | 1.027600446 | 2.75E-19 | 1.51E-18 |
| GALE | 10.83432624 | 18.55155752 | 0.775930877 | 2.02E-11 | 4.99E-11 |
| PYCR2 | 5.37894786 | 15.26388831 | 1.50472661 | 5.48E-28 | 4.93E-26 |
| GUCY2D | 0.031734767 | 0.213838028 | 2.752382262 | 2.84E-05 | 4.23E-05 |
| SULT1A1 | 13.47559808 | 9.050285627 | -0.57431408 | 3.86E-09 | 7.64E-09 |
| MDH2 | 46.934258 | 79.50191043 | 0.760348177 | 6.89E-16 | 2.43E-15 |
| ACYP1 | 0.435832892 | 1.541828841 | 1.822795635 | 4.19E-26 | 1.54E-24 |
| AKR1C1 | 27.5295606 | 64.19352837 | 1.221446277 | 0.000384398 | 0.000518074 |
| ACO2 | 12.52959346 | 20.83091178 | 0.733386383 | 3.46E-08 | 6.38E-08 |
| PFAS | 1.153738786 | 2.725413061 | 1.240158275 | 5.61E-19 | 2.93E-18 |
| HMBS | 2.84492594 | 6.077665142 | 1.095126093 | 4.39E-23 | 5.00E-22 |
| ACP4 | 0.013881318 | 0.395615568 | 4.83288278 | 1.58E-12 | 4.22E-12 |
| ACSS1 | 0.750883248 | 2.273225868 | 1.598080527 | 1.27E-05 | 1.96E-05 |
| POLR3G | 0.27411095 | 0.623002221 | 1.184477344 | 3.97E-13 | 1.15E-12 |
| ENTPD3 | 0.01130905 | 0.150983176 | 3.738838219 | 0.00084192 | 0.001107071 |
| NME7 | 0.643526996 | 1.362272741 | 1.081942999 | 4.98E-17 | 1.96E-16 |
| ADA | 0.943392724 | 2.67245149 | 1.502233382 | 1.19E-17 | 5.20E-17 |
| POLR1C | 3.35005178 | 6.978792797 | 1.058794103 | 2.33E-21 | 1.79E-20 |
| ALOX12 | 0.109011063 | 0.275266308 | 1.336353479 | 1.04E-12 | 2.85E-12 |
| JMJD7-PLA2G4B | 0.08957713 | 0.24466282 | 1.449592525 | 1.59E-10 | 3.62E-10 |
| TAZ | 1.723546396 | 5.52082373 | 1.679503404 | 7.52E-29 | 2.03E-26 |
| NT5C3A | 1.954837404 | 4.189451043 | 1.099712601 | 2.24E-17 | 9.32E-17 |
| CHST11 | 0.731928728 | 1.923006546 | 1.393588597 | 0.008023001 | 0.009699449 |
| ENPP2 | 4.152915006 | 9.060232331 | 1.1254237 | 9.16E-05 | 0.000131551 |
| INPPL1 | 4.00731938 | 9.917278334 | 1.30730675 | 4.82E-21 | 3.39E-20 |
| NT5M | 0.40577981 | 1.573026546 | 1.954774029 | 3.24E-17 | 1.31E-16 |
| CRLS1 | 14.10303008 | 21.11234898 | 0.58208194 | 0.000158942 | 0.00022197 |
| ACSM5 | 50.4663964 | 20.22345997 | -1.31929323 | 2.44E-17 | 1.00E-16 |
| ALOX15B | 0.142066661 | 1.817887709 | 3.677623143 | 7.72E-07 | 1.32E-06 |
| WARS | 6.03011432 | 11.16265189 | 0.888422547 | 1.69E-08 | 3.17E-08 |
| AK1 | 2.37631508 | 3.937539076 | 0.728568102 | 1.41E-08 | 2.65E-08 |
| UGT1A6 | 2.955261072 | 7.241389897 | 1.292981046 | 0.006980058 | 0.008463846 |
| ITPA | 8.35692768 | 20.76255349 | 1.312939329 | 2.62E-22 | 2.47E-21 |
| GANC | 0.61022308 | 1.019814907 | 0.740898679 | 3.01E-10 | 6.63E-10 |
| MTMR1 | 1.628000482 | 3.071386948 | 0.915789155 | 1.92E-14 | 6.17E-14 |
| PLCD1 | 1.691310116 | 2.545895876 | 0.590032202 | 2.56E-07 | 4.48E-07 |
| EPRS | 6.85059394 | 18.77455891 | 1.454478035 | 1.37E-25 | 3.41E-24 |
| ALDH3A1 | 0.752936723 | 44.07999412 | 5.871451597 | 0.000343499 | 0.000465275 |
| ME1 | 2.644506866 | 8.410134567 | 1.669130164 | 0.000433795 | 0.000580784 |
| GSTA4 | 3.88265552 | 12.89632448 | 1.73184433 | 2.91E-16 | 1.07E-15 |
| ADCY4 | 0.314709281 | 0.7262269 | 1.206400646 | 6.53E-13 | 1.83E-12 |
| POLR2E | 23.3947028 | 33.18577334 | 0.504382993 | 1.39E-11 | 3.50E-11 |
| PGM2L1 | 0.22568328 | 0.446572794 | 0.984595823 | 0.001355076 | 0.001750577 |
| LYPLA2 | 21.932233 | 34.59496813 | 0.657509506 | 4.98E-13 | 1.42E-12 |
| POLR3K | 2.48922156 | 5.518207323 | 1.148505014 | 2.44E-21 | 1.86E-20 |
| EPHX2 | 46.3273598 | 24.00327051 | -0.94863347 | 3.57E-17 | 1.42E-16 |
| HCCS | 5.76547254 | 8.626777767 | 0.581382935 | 3.21E-10 | 7.05E-10 |
| DHDH | 0.083578928 | 0.534516557 | 2.677023475 | 3.88E-10 | 8.42E-10 |
| DGKQ | 1.457440362 | 4.03086916 | 1.467654105 | 4.12E-23 | 4.77E-22 |
| UROS | 3.93219928 | 6.103738102 | 0.634356623 | 8.59E-11 | 2.00E-10 |
| SGPL1 | 5.76193592 | 8.91802992 | 0.630171424 | 5.88E-09 | 1.14E-08 |
| GART | 3.11647116 | 5.301358952 | 0.766448866 | 3.40E-14 | 1.07E-13 |
| ARG1 | 240.2322 | 166.3619414 | -0.53010411 | 1.68E-08 | 3.16E-08 |
| MIOX | 0.025307544 | 0.730525513 | 4.851295268 | 1.25E-11 | 3.16E-11 |
| GNE | 25.03165064 | 13.02163252 | -0.9428431 | 2.36E-17 | 9.77E-17 |
| ACER3 | 0.859076874 | 1.427533057 | 0.732665013 | 9.70E-08 | 1.73E-07 |
| CYP3A4 | 765.778506 | 344.3796666 | -1.1529272 | 1.69E-15 | 5.81E-15 |
| UMPS | 3.1273573 | 4.722024104 | 0.59446135 | 3.46E-13 | 1.01E-12 |
| AGXT2 | 21.68184318 | 9.089264569 | -1.25425193 | 2.47E-18 | 1.19E-17 |
| INPP5A | 3.35817814 | 4.788868444 | 0.512006041 | 1.30E-07 | 2.30E-07 |
| ACSM3 | 8.26916818 | 3.080383675 | -1.42463216 | 3.51E-21 | 2.59E-20 |
| PAFAH1B3 | 2.183462234 | 13.95513656 | 2.676106759 | 2.23E-19 | 1.24E-18 |
| UGT2B10 | 198.5524482 | 92.80896422 | -1.09718409 | 9.18E-13 | 2.53E-12 |
| POLR2L | 45.7742558 | 93.97066345 | 1.037674004 | 3.18E-19 | 1.72E-18 |
| ADSL | 3.82300396 | 8.444661914 | 1.143332971 | 6.03E-24 | 8.42E-23 |
| NADSYN1 | 1.32031081 | 2.690367867 | 1.026925864 | 1.07E-21 | 9.08E-21 |
| CAD | 1.068638102 | 4.256222464 | 1.993800198 | 5.58E-26 | 1.88E-24 |
| PIK3C2G | 2.247678282 | 1.473184304 | -0.60949762 | 2.52E-08 | 4.69E-08 |
| POLR2D | 3.37104148 | 5.942900711 | 0.817972898 | 1.73E-20 | 1.09E-19 |
| ACAT1 | 82.9427028 | 47.2412729 | -0.81206722 | 2.12E-16 | 7.92E-16 |
| UROC1 | 40.84584802 | 11.96254955 | -1.77166454 | 3.22E-19 | 1.73E-18 |
| PGP | 2.46275828 | 8.344557131 | 1.76056045 | 1.37E-25 | 3.41E-24 |
| NEU1 | 7.53752836 | 26.66679468 | 1.822880995 | 3.40E-28 | 4.21E-26 |
| TXNDC12 | 10.49130104 | 17.80804426 | 0.763335484 | 7.64E-21 | 5.15E-20 |
| TYMP | 15.65976866 | 31.83914656 | 1.023738765 | 1.52E-06 | 2.51E-06 |
| PCK1 | 310.8995322 | 98.70077197 | -1.65531517 | 1.33E-19 | 7.63E-19 |
| GNPAT | 8.1486725 | 23.37881262 | 1.520564705 | 1.98E-27 | 1.46E-25 |
| AMY2B | 0.407575554 | 0.944786186 | 1.212920347 | 3.39E-06 | 5.50E-06 |
| CYP2C18 | 28.99648586 | 18.82865448 | -0.62294816 | 2.50E-09 | 5.03E-09 |
| CP | 186.4100454 | 97.23571025 | -0.93892145 | 7.95E-13 | 2.20E-12 |
| SCLY | 0.20643543 | 0.46320077 | 1.165947056 | 1.86E-14 | 6.02E-14 |
| ACACA | 1.393724136 | 3.919558477 | 1.491746117 | 6.37E-20 | 3.74E-19 |
| CYP4A22 | 60.5277696 | 19.74899941 | -1.61581763 | 1.17E-20 | 7.56E-20 |
| DGKZ | 1.274013736 | 3.212991644 | 1.334536397 | 1.11E-23 | 1.50E-22 |
| POLD1 | 1.184827396 | 4.730074491 | 1.997185999 | 1.56E-26 | 8.44E-25 |
| NUDT2 | 7.28607756 | 17.12103503 | 1.232555663 | 4.35E-19 | 2.29E-18 |
| ACAA1 | 48.7472452 | 25.20723697 | -0.9514827 | 4.88E-18 | 2.28E-17 |
| ZNRD1 | 1.920201268 | 4.468929856 | 1.218671864 | 4.96E-20 | 2.98E-19 |
| PIK3CB | 1.966956368 | 3.395752372 | 0.787766302 | 5.26E-13 | 1.49E-12 |
| POLR2G | 8.5867008 | 20.36548606 | 1.245950419 | 3.97E-26 | 1.53E-24 |
| UGT1A10 | 0.008250359 | 0.550736449 | 6.060761396 | 0.01163501 | 0.013879762 |
| GALK1 | 24.967336 | 42.45332342 | 0.765835604 | 9.54E-05 | 0.000136769 |
| PLD2 | 1.824123198 | 3.033992898 | 0.734014539 | 8.11E-09 | 1.56E-08 |
| SPTLC2 | 1.75140612 | 2.742051601 | 0.646742062 | 3.29E-10 | 7.20E-10 |
| RDH5 | 4.71202044 | 2.420467525 | -0.96106006 | 6.15E-15 | 2.06E-14 |
| UPP1 | 2.553424264 | 3.724478367 | 0.544605114 | 0.000545856 | 0.000723638 |
| PLA2G10 | 0.026018179 | 0.05980117 | 1.200653711 | 0.036085682 | 0.04140142 |
| MTHFD1L | 0.557753522 | 2.524923016 | 2.178539778 | 2.35E-22 | 2.24E-21 |
| PHOSPHO1 | 0.134396765 | 0.094790887 | -0.50367814 | 3.82E-07 | 6.65E-07 |
| PDE6D | 2.1416811 | 4.452477304 | 1.055864581 | 5.61E-23 | 6.22E-22 |
| AHCY | 44.0853498 | 69.89104425 | 0.664808295 | 2.94E-08 | 5.45E-08 |
| AC005759.1 | 0.035657874 | 0.074263706 | 1.058436633 | 0.007357208 | 0.00890783 |
| NMNAT1 | 1.26411244 | 1.805223587 | 0.514052741 | 9.85E-06 | 1.53E-05 |
| GUK1 | 19.8499378 | 43.05143148 | 1.116926722 | 8.60E-22 | 7.57E-21 |
| GSR | 15.11395882 | 25.70655358 | 0.766254607 | 4.11E-05 | 6.07E-05 |
| G6PD | 1.311714874 | 13.58214647 | 3.372185434 | 6.03E-25 | 1.25E-23 |
| SMS | 10.69932606 | 20.11237342 | 0.910563416 | 7.48E-17 | 2.89E-16 |
| CPS1 | 205.3781916 | 136.0158919 | -0.59450777 | 2.45E-07 | 4.29E-07 |
| HYI | 3.01594442 | 5.084056814 | 0.753370311 | 2.55E-11 | 6.22E-11 |
| OGDH | 13.79518708 | 24.98405926 | 0.856842875 | 4.59E-13 | 1.32E-12 |
| PI4KA | 2.861807906 | 5.047843187 | 0.818740256 | 2.57E-10 | 5.72E-10 |
| ALDH2 | 154.9258366 | 71.34767851 | -1.11863937 | 1.47E-22 | 1.45E-21 |
| LRAT | 1.094300194 | 0.233448683 | -2.22883121 | 9.54E-25 | 1.80E-23 |
| POLE3 | 8.66220048 | 16.03828744 | 0.888714631 | 6.27E-19 | 3.25E-18 |
| NANP | 0.94921358 | 1.727478428 | 0.863863049 | 2.01E-15 | 6.85E-15 |
| CPT1B | 0.121235197 | 0.398590265 | 1.717097872 | 1.74E-14 | 5.65E-14 |
| FTH1 | 139.0992968 | 286.4863544 | 1.042351297 | 8.93E-17 | 3.41E-16 |
| POLE | 1.3055207 | 2.220828061 | 0.766472369 | 6.30E-10 | 1.34E-09 |
| INPP5E | 1.353489428 | 3.225346035 | 1.252770331 | 1.93E-19 | 1.08E-18 |
| GAL3ST1 | 0.673563029 | 6.610136193 | 3.294795138 | 0.00012214 | 0.000172357 |
| FLAD1 | 4.34967458 | 13.71639311 | 1.656921782 | 1.85E-29 | 7.49E-27 |
| ITPKA | 0.460224664 | 4.393506423 | 3.254962596 | 5.55E-18 | 2.57E-17 |
| NME1 | 5.12519878 | 17.66287993 | 1.785040727 | 4.09E-24 | 6.02E-23 |
| ITPK1 | 7.76110164 | 15.32881022 | 0.981912369 | 6.29E-16 | 2.23E-15 |
| POLR3A | 1.148311722 | 2.372790232 | 1.047070233 | 7.34E-23 | 7.82E-22 |
| PFKFB2 | 0.382136054 | 1.420710904 | 1.89445473 | 6.30E-20 | 3.72E-19 |
| CYP2B6 | 126.8640398 | 29.24442074 | -2.11704987 | 1.14E-20 | 7.45E-20 |
| ENTPD4 | 1.775126334 | 2.540169076 | 0.517002824 | 0.002229478 | 0.002834972 |
| ALDH18A1 | 7.39242978 | 15.6829565 | 1.085077018 | 4.28E-15 | 1.45E-14 |
| PAFAH1B2 | 5.55860366 | 8.178248219 | 0.557069332 | 2.81E-10 | 6.21E-10 |
| LDHD | 55.065139 | 32.56811545 | -0.75767902 | 6.24E-13 | 1.75E-12 |
| FBP1 | 394.352886 | 138.9132655 | -1.50530283 | 4.76E-21 | 3.38E-20 |
| IDO2 | 0.862516082 | 0.34741407 | -1.31189517 | 9.61E-15 | 3.18E-14 |
| AANAT | 0.01054337 | 0.053242243 | 2.336235303 | 3.58E-10 | 7.82E-10 |
| MPI | 2.65308182 | 4.29403827 | 0.694665881 | 6.65E-13 | 1.86E-12 |
| GFPT1 | 4.77169852 | 8.360365723 | 0.809063159 | 2.23E-11 | 5.47E-11 |
| PIK3C2A | 2.259323538 | 3.277646011 | 0.536769169 | 3.36E-05 | 4.99E-05 |
| MIF | 18.5365111 | 45.69842886 | 1.301774837 | 2.78E-13 | 8.17E-13 |
| GMPS | 2.12571058 | 5.125880879 | 1.269854768 | 3.78E-23 | 4.51E-22 |
| DGKI | 0.004675862 | 0.038436959 | 3.039190001 | 4.91E-25 | 1.07E-23 |
| ALDH1A1 | 267.4815334 | 396.7508063 | 0.568793862 | 0.021467023 | 0.024983173 |
| PDE6A | 0.003914199 | 0.018007188 | 2.201783842 | 1.90E-08 | 3.55E-08 |
| GGT7 | 3.07695892 | 5.573133026 | 0.856983407 | 2.99E-11 | 7.16E-11 |
| LDHAL6A | 0.009471548 | 0.024815681 | 1.38957983 | 0.000628518 | 0.000829153 |
| PLCB3 | 1.634257378 | 3.846683794 | 1.234980031 | 1.86E-21 | 1.46E-20 |
| AGL | 6.99793654 | 3.850530943 | -0.86187219 | 9.11E-17 | 3.47E-16 |
| PTGS2 | 0.685695488 | 0.209820152 | -1.70841478 | 5.31E-17 | 2.08E-16 |
| PNPT1 | 2.55535368 | 4.267066066 | 0.739721463 | 3.15E-14 | 9.96E-14 |
| GAD1 | 0.002933251 | 0.132776766 | 5.500358612 | 4.82E-14 | 1.49E-13 |
| CDA | 29.0720696 | 17.30167458 | -0.7487221 | 1.20E-10 | 2.78E-10 |
| OXCT1 | 0.275234787 | 1.493923329 | 2.440371378 | 0.000229146 | 0.000315124 |
| EARS2 | 3.1501315 | 5.085392201 | 0.690946989 | 1.33E-11 | 3.36E-11 |
| ENTPD6 | 4.89225452 | 13.77275387 | 1.493245689 | 7.12E-25 | 1.41E-23 |
| SARDH | 34.544682 | 18.5565624 | -0.89653416 | 1.76E-17 | 7.58E-17 |
| GATM | 201.0644772 | 134.4996308 | -0.580056 | 1.14E-08 | 2.18E-08 |
| PIP5K1A | 3.47425607 | 7.244620632 | 1.060206053 | 1.21E-14 | 3.99E-14 |
| METTL6 | 0.430309778 | 1.185576642 | 1.462141401 | 6.71E-28 | 5.44E-26 |
| UGT8 | 0.017681348 | 0.163356071 | 3.207719889 | 0.009538855 | 0.011514862 |
| PFKL | 12.61516374 | 19.37322019 | 0.618904845 | 2.04E-10 | 4.61E-10 |
| PLCB4 | 0.052123637 | 0.196713256 | 1.916084508 | 4.58E-08 | 8.38E-08 |
| MTMR7 | 0.146421709 | 0.848734424 | 2.535183728 | 1.88E-05 | 2.86E-05 |
| PDE7A | 0.574264186 | 1.45451893 | 1.340755577 | 3.24E-14 | 1.02E-13 |
| PLA2G2C | 0.005939526 | 0.01860975 | 1.647638864 | 1.36E-05 | 2.09E-05 |
| GPD1 | 36.7540008 | 17.69852929 | -1.05427182 | 4.63E-13 | 1.33E-12 |
| SMPD2 | 1.62738852 | 3.829329681 | 1.234533154 | 1.20E-19 | 6.95E-19 |
| EHHADH | 68.1731674 | 41.91216309 | -0.70183503 | 2.39E-10 | 5.35E-10 |
| NPL | 2.542158828 | 4.829610362 | 0.925852632 | 0.012594123 | 0.014914093 |
| GSS | 17.1787072 | 29.50461679 | 0.780319251 | 4.06E-18 | 1.91E-17 |
| AMPD1 | 0.080093498 | 0.027934586 | -1.51963269 | 1.41E-12 | 3.79E-12 |
| GLB1 | 9.81417212 | 18.17416942 | 0.888950954 | 8.00E-18 | 3.64E-17 |
| POLR3D | 0.963096128 | 1.733721133 | 0.848120154 | 5.64E-08 | 1.02E-07 |
| NAGK | 2.34741402 | 3.529596543 | 0.588430966 | 4.54E-10 | 9.75E-10 |
| PCYT1A | 3.16719152 | 5.454631679 | 0.784277673 | 2.39E-20 | 1.46E-19 |
| CA2 | 43.516094 | 27.05355184 | -0.68573105 | 1.63E-13 | 4.87E-13 |
| OCRL | 1.958109446 | 5.65422446 | 1.529867751 | 1.85E-24 | 3.12E-23 |
| ME2 | 1.285344598 | 2.315627387 | 0.84924793 | 4.85E-09 | 9.59E-09 |
| SULT1A3 | 0.00416242 | 0.017221506 | 2.048716894 | 1.82E-09 | 3.71E-09 |
| FADS2 | 9.642111654 | 20.05759983 | 1.056727937 | 0.011186336 | 0.013383947 |
| HAO2 | 86.8433924 | 25.34855366 | -1.77651265 | 1.58E-20 | 9.97E-20 |
| BPNT1 | 5.53523458 | 10.96770642 | 0.986545495 | 3.39E-21 | 2.52E-20 |
| ALDH3B1 | 1.04183392 | 2.518889866 | 1.27366273 | 1.40E-06 | 2.33E-06 |
| TYMS | 1.561858024 | 8.89844653 | 2.51029018 | 1.44E-20 | 9.16E-20 |
| HEXB | 14.85885542 | 30.48633278 | 1.036839629 | 3.86E-21 | 2.81E-20 |
| ACADS | 72.100922 | 32.05868751 | -1.16930235 | 9.35E-22 | 8.14E-21 |
| POLR1D | 4.90762592 | 8.660998203 | 0.819508024 | 2.72E-18 | 1.30E-17 |
| GPX7 | 1.50203363 | 4.344910673 | 1.532409402 | 0.000302397 | 0.000411667 |
| GNMT | 134.7991696 | 71.11434267 | -0.92259915 | 2.73E-11 | 6.59E-11 |
| DHODH | 15.80542924 | 6.798435324 | -1.21714557 | 4.99E-16 | 1.79E-15 |
| PLA2G15 | 2.72944324 | 4.31619806 | 0.661154372 | 5.77E-08 | 1.04E-07 |
| MAT2A | 11.17323804 | 17.09991339 | 0.613941674 | 0.000105036 | 0.000150316 |
| XDH | 15.64604762 | 9.530809199 | -0.71512765 | 1.12E-09 | 2.32E-09 |
| AACS | 0.31328518 | 1.03790029 | 1.728119422 | 1.45E-19 | 8.24E-19 |
| ALLC | 0.155015949 | 0.106478453 | -0.54185514 | 1.43E-13 | 4.29E-13 |
| LCMT1 | 2.3080183 | 5.778087404 | 1.323937364 | 5.58E-25 | 1.19E-23 |
| CERK | 5.53242282 | 11.22309283 | 1.02048698 | 1.28E-14 | 4.19E-14 |
| NT5C2 | 1.540448554 | 2.299890391 | 0.578214605 | 0.001724337 | 0.002213491 |
| BDH2 | 10.50709572 | 6.470275333 | -0.69946494 | 1.71E-12 | 4.54E-12 |
