# Supplementary material for: Six Metabolism Related mRNAs Predict the Prognosis of Patients With Hepatocellular Carcinoma
Source: Front Mol Biosci. 2021 Feb 25;8:621232. doi: 10.3389/fmolb.2021.621232 (PMC8045485; doi:10.3389/fmolb.2021.621232)
Supplement: Supplementary file 1 [file datasheet1.zip › Supplementary tables/Supplementary table 2 and 3.docx]

2. Supplementary table 2: The Hazard ratio (HR), HR.95L and HR.95H

value of 45 differentially expressed genes’ mRNAs

| id | HR | HR.95L | HR.95H | p-value |
| --- | --- | --- | --- | --- |
| MARS | 1.109907687 | 1.054750096 | 1.167949715 | 6.08E-05 |
| LCLAT1 | 1.493555674 | 1.205747795 | 1.850062311 | 0.000239644 |
| POLR3F | 1.462513164 | 1.223370619 | 1.748402914 | 3.00E-05 |
| DLAT | 1.068347772 | 1.034086659 | 1.103744017 | 7.02E-05 |
| DNMT1 | 1.119433412 | 1.046691144 | 1.197231077 | 0.000997785 |
| FPGT | 1.305478348 | 1.118712401 | 1.523424354 | 0.000714261 |
| GPD2 | 1.373330819 | 1.140795733 | 1.653264897 | 0.000803277 |
| PRIM1 | 1.197847518 | 1.112700552 | 1.289510168 | 1.60E-06 |
| UGDH | 1.011038824 | 1.005413264 | 1.016695861 | 0.000115095 |
| PGD | 1.010033369 | 1.005602229 | 1.014484034 | 8.57E-06 |
| POLA1 | 1.50408186 | 1.254757648 | 1.802947561 | 1.01E-05 |
| ACP1 | 1.046627426 | 1.018687278 | 1.075333904 | 0.000963111 |
| GPD1L | 1.232655393 | 1.101364157 | 1.379597572 | 0.000272393 |
| DCK | 1.126472371 | 1.052725086 | 1.205385926 | 0.000566178 |
| ATIC | 1.050730185 | 1.024727702 | 1.077392481 | 0.000108597 |
| AGPS | 1.143330667 | 1.063646772 | 1.22898414 | 0.000279058 |
| MBOAT7 | 1.068990227 | 1.028512928 | 1.111060517 | 0.000705405 |
| PSPH | 1.043555738 | 1.021142344 | 1.066461092 | 0.000118787 |
| PLCB1 | 1.45414434 | 1.169445932 | 1.808151795 | 0.000757159 |
| UCK2 | 1.152769032 | 1.101721918 | 1.20618136 | 7.65E-10 |
| POLR1A | 1.296563404 | 1.116740334 | 1.505342478 | 0.000651026 |
| SEPHS1 | 1.0889111 | 1.052646592 | 1.12642495 | 8.27E-07 |
| POLR3C | 1.098005217 | 1.041937993 | 1.157089448 | 0.000471872 |
| GNPDA1 | 1.068964224 | 1.029761896 | 1.109658957 | 0.000468005 |
| RRM1 | 1.05191576 | 1.027106177 | 1.077324614 | 3.24E-05 |
| DTYMK | 1.048476628 | 1.024674314 | 1.07283185 | 5.34E-05 |
| TXNRD1 | 1.015784348 | 1.009809051 | 1.021795004 | 1.96E-07 |
| RRM2 | 1.075624185 | 1.037508679 | 1.115139961 | 7.48E-05 |
| PPAT | 1.563187243 | 1.28959852 | 1.894817896 | 5.34E-06 |
| POLR3G | 2.128068517 | 1.417536761 | 3.194750031 | 0.000269313 |
| HCCS | 1.104537282 | 1.046390936 | 1.165914731 | 0.000314027 |
| GART | 1.189895018 | 1.077201396 | 1.314378313 | 0.000615113 |
| CAD | 1.189082437 | 1.103198204 | 1.281652777 | 5.96E-06 |
| ACACA | 1.192736463 | 1.085703054 | 1.310321698 | 0.000238728 |
| POLD1 | 1.12350823 | 1.051765678 | 1.200144451 | 0.000542033 |
| UGT1A10 | 1.130879502 | 1.056337331 | 1.210681864 | 0.000407273 |
| GSR | 1.017864917 | 1.007737207 | 1.028094409 | 0.000519249 |
| G6PD | 1.011375463 | 1.00619009 | 1.01658756 | 1.61E-05 |
| SMS | 1.055498465 | 1.03457978 | 1.076840115 | 1.23E-07 |
| POLR3A | 1.681844328 | 1.242627559 | 2.276305821 | 0.000760802 |
| GMPS | 1.236958121 | 1.132302539 | 1.351286727 | 2.42E-06 |
| METTL6 | 2.834338226 | 1.7357204 | 4.628322153 | 3.13E-05 |
| TYMS | 1.049925665 | 1.0236666 | 1.076858327 | 0.000163252 |
| HEXB | 1.032201666 | 1.013703213 | 1.051037687 | 0.000592425 |
| AACS | 1.951244147 | 1.397165261 | 2.725056103 | 8.77E-05 |

3. Supplementary table3: The correlation coefficient of each mRNA

| Gene | Coefficient |
| --- | --- |
| PRIM1 | 0.003628573 |
| UCK2 | 0.049799945 |
| SEPHS1 | 0.008740925 |
| TXNRD1 | 0.002877531 |
| SMS | 0.010921528 |
| GMPS | 0.020683567 |
