# Supplementary material for: Six Metabolism Related mRNAs Predict the Prognosis of Patients With Hepatocellular Carcinoma
Source: Front Mol Biosci. 2021 Feb 25;8:621232. doi: 10.3389/fmolb.2021.621232 (PMC8045485; doi:10.3389/fmolb.2021.621232)
Supplement: Supplementary file 1 [file datasheet1.zip › Supplementary tables/Supplementary table 4.docx]

Supplementary table 4: The overall grouping information for training cohort and validation cohort.

| Training cohort | |  |  |  |  |  |  |  |  |  |
| --- | --- | --- | --- | --- | --- | --- | --- | --- | --- | --- |
| Id | futime | fustat | PRIM1 | UCK2 | SEPHS1 | TXNRD1 | SMS | GMPS | riskScore | risk |
| TCGA-RC-A6M3 | 0.00274 | 0 | 5.5731 | 9.3965 | 15.335 | 13.046 | 23.972 | 9.29 | 1.113711581 | high |
| TCGA-BW-A5NP | 0.00274 | 0 | 4.0246 | 8.1789 | 18.463 | 13.591 | 19.288 | 8.3706 | 1.006186469 | high |
| TCGA-ED-A97K | 0.01644 | 0 | 1.9436 | 5.6527 | 7.7223 | 17.375 | 56.16 | 5.5339 | 1.133867899 | high |
| TCGA-BC-A3KF | 0.02192 | 0 | 3.7522 | 2.7347 | 11.491 | 13.993 | 18.979 | 3.6329 | 0.572923249 | low |
| TCGA-DD-AADM | 0.03288 | 1 | 3.0786 | 5.5902 | 10.581 | 29.203 | 17.956 | 4.7182 | 0.759771068 | high |
| TCGA-DD-A11B | 0.03836 | 1 | 1.7469 | 3.7031 | 10.734 | 9.4636 | 14.916 | 4.6824 | 0.571559975 | low |
| TCGA-DD-AAC8 | 0.04384 | 1 | 6.0055 | 10.779 | 8.847 | 94.43 | 21.332 | 8.0266 | 1.306651808 | high |
| TCGA-BW-A5NO | 0.05479 | 0 | 5.5517 | 2.9762 | 17.582 | 18.013 | 14.309 | 3.9709 | 0.612286648 | low |
| TCGA-ZP-A9D1 | 0.05753 | 0 | 1.4214 | 4.7705 | 10.829 | 37.523 | 21.505 | 3.9785 | 0.762514238 | high |
| TCGA-RC-A6M4 | 0.06027 | 0 | 4.3807 | 3.9235 | 10.2 | 18.554 | 10.546 | 3.8475 | 0.548587846 | low |
| TCGA-WQ-A9G7 | 0.08219 | 0 | 1.0264 | 6.8406 | 12.193 | 5.7931 | 24.236 | 4.7021 | 0.829579925 | high |
| TCGA-BC-A10Z | 0.09315 | 1 | 5.92 | 5.7047 | 19.795 | 21.733 | 14.142 | 10.104 | 0.904581212 | high |
| TCGA-UB-A7MD | 0.14247 | 1 | 2.9484 | 4.9939 | 12.456 | 112.22 | 26.268 | 5.8845 | 1.09980094 | high |
| TCGA-KR-A7K0 | 0.17808 | 1 | 1.5484 | 3.9869 | 12.274 | 86.134 | 24.053 | 4.0218 | 0.905183988 | high |
| TCGA-G3-AAV6 | 0.17808 | 1 | 6.2932 | 7.6287 | 22.992 | 6.8026 | 24.533 | 6.5239 | 1.026166515 | high |
| TCGA-DD-A11A | 0.21644 | 0 | 3.3875 | 4.0105 | 13.091 | 34.16 | 7.1085 | 4.6258 | 0.598054842 | low |
| TCGA-CC-5260 | 0.23836 | 1 | 2.9647 | 10.968 | 23.006 | 8.7235 | 32.053 | 11.146 | 1.36378288 | high |
| TCGA-BC-A10W | 0.24932 | 1 | 12.373 | 33.551 | 10.925 | 178.53 | 21.028 | 12.413 | 2.81136801 | high |
| TCGA-QA-A7B7 | 0.25753 | 0 | 17.019 | 7.4379 | 9.017 | 13.204 | 19.841 | 9.7029 | 0.966360121 | high |
| TCGA-CC-5261 | 0.26575 | 1 | 5.8876 | 5.8527 | 18.659 | 12.457 | 25.541 | 10.785 | 1.013778866 | high |
| TCGA-CC-A1HT | 0.27671 | 1 | 6.2237 | 11.986 | 20.9 | 138.63 | 47.425 | 9.6961 | 1.919628183 | high |
| TCGA-CC-5264 | 0.27945 | 1 | 11.162 | 5.7128 | 28.621 | 13.604 | 23.514 | 9.0287 | 1.057869804 | high |
| TCGA-CC-5262 | 0.28219 | 1 | 2.4818 | 7.0421 | 16.788 | 47.869 | 27.222 | 6.1265 | 1.068213792 | high |
| TCGA-DD-AADF | 0.31507 | 1 | 1.8732 | 4.2618 | 19.031 | 8.3188 | 22.686 | 4.1103 | 0.742104482 | high |
| TCGA-CC-5258 | 0.35342 | 1 | 9.8115 | 11.081 | 16.099 | 67.634 | 30.56 | 12.505 | 1.515174046 | high |
| TCGA-CC-A8HT | 0.38356 | 1 | 5.3366 | 9.8077 | 27.019 | 91.859 | 34.74 | 7.7329 | 1.547633608 | high |
| TCGA-DD-AACZ | 0.46849 | 1 | 7.9876 | 11.893 | 20.644 | 139.77 | 41.488 | 10.833 | 1.881044889 | high |
| TCGA-FV-A3R2 | 0.53151 | 1 | 3.3748 | 4.4463 | 15.967 | 22.566 | 18.86 | 7.0974 | 0.79094539 | high |
| TCGA-DD-AACH | 0.53425 | 1 | 16.047 | 17.108 | 13.845 | 155.95 | 24.437 | 11.642 | 1.987646915 | high |
| TCGA-CC-A9FS | 0.57808 | 0 | 3.0144 | 4.0768 | 14.057 | 8.5245 | 11.246 | 7.084 | 0.630712481 | low |
| TCGA-UB-A7MF | 0.5863 | 1 | 3.9369 | 3.4551 | 18.791 | 13.441 | 21.155 | 7.8915 | 0.783541364 | high |
| TCGA-CC-A123 | 0.6 | 0 | 2.5432 | 3.8323 | 14.502 | 2.263 | 21.572 | 4.2156 | 0.656144453 | low |
| TCGA-DD-A119 | 0.61096 | 1 | 1.0501 | 3.0276 | 7.0804 | 21.669 | 25.667 | 3.3966 | 0.629403202 | low |
| TCGA-MR-A520 | 0.6274 | 0 | 1.0701 | 0.9622 | 10.579 | 6.0804 | 9.4016 | 2.1669 | 0.309270361 | low |
| TCGA-EP-A26S | 0.64932 | 0 | 3.6596 | 3.9406 | 20.395 | 17.051 | 15.548 | 5.0188 | 0.710476671 | high |
| TCGA-FV-A3I1 | 0.67671 | 1 | 3.9606 | 4.8842 | 16.163 | 71.839 | 20.952 | 4.4537 | 0.926544192 | high |
| TCGA-4R-AA8I | 0.71781 | 1 | 3.6721 | 1.751 | 10.257 | 30.993 | 11.565 | 3.4627 | 0.477294494 | low |
| TCGA-CC-A7IK | 0.71781 | 1 | 8.0124 | 10.191 | 14.222 | 167.38 | 14.896 | 7.6493 | 1.463430835 | high |
| TCGA-CC-A5UE | 0.74521 | 1 | 13.408 | 10.682 | 18.88 | 85.731 | 32.449 | 5.0915 | 1.452028261 | high |
| TCGA-DD-A73B | 0.77534 | 1 | 5.8853 | 2.8867 | 15.629 | 8.6638 | 20.415 | 10.28 | 0.762237055 | high |
| TCGA-MI-A75C | 0.79726 | 0 | 3.6358 | 13.421 | 10.392 | 92.991 | 15.789 | 3.333 | 1.281358758 | high |
| TCGA-ED-A66Y | 0.81096 | 1 | 10.103 | 5.4503 | 12.721 | 3.9222 | 17.526 | 7.0071 | 0.766905875 | high |
| TCGA-CC-A7IG | 0.81918 | 1 | 6.8659 | 11.161 | 19.777 | 10.99 | 17.696 | 8.5713 | 1.15575762 | high |
| TCGA-CC-A8HS | 0.82192 | 1 | 4.7858 | 9.9842 | 23.437 | 9.1624 | 20.657 | 10.639 | 1.191457581 | high |
| TCGA-EP-A3JL | 0.83014 | 0 | 2.3916 | 6.3051 | 12.797 | 44.912 | 32.663 | 2.9829 | 0.982200158 | high |
| TCGA-UB-AA0V | 0.86027 | 0 | 2.2436 | 1.4102 | 10.782 | 8.2127 | 12.874 | 3.1173 | 0.401321032 | low |
| TCGA-5C-A9VH | 0.88219 | 0 | 3.2475 | 3.716 | 11.518 | 7.8951 | 20.102 | 5.6 | 0.655610889 | low |
| TCGA-UB-AA0U | 0.89589 | 0 | 6.3873 | 5.5164 | 15.983 | 16.568 | 13.543 | 6.2465 | 0.762391249 | high |
| TCGA-MR-A8JO | 0.90411 | 0 | 0.8881 | 4.6035 | 13.109 | 13.674 | 20.24 | 3.381 | 0.677393649 | low |
| TCGA-EP-A2KB | 0.91507 | 0 | 5.4881 | 6.4288 | 15.443 | 6.646 | 18.52 | 5.5704 | 0.811651337 | high |
| TCGA-CC-A8HU | 0.94247 | 1 | 8.773 | 6.6911 | 39.56 | 15.254 | 65.921 | 8.7161 | 1.654975458 | high |
| TCGA-CC-A5UC | 0.95068 | 1 | 7.0606 | 10.494 | 18.147 | 41.108 | 38.121 | 8.0058 | 1.407038322 | high |
| TCGA-BC-4073 | 0.96438 | 0 | 4.673 | 3.9011 | 11.378 | 36.946 | 15.216 | 7.1991 | 0.732083565 | high |
| TCGA-G3-AAV5 | 0.96986 | 0 | 3.6813 | 11.52 | 16.813 | 94.279 | 21.29 | 4.7703 | 1.336475915 | high |
| TCGA-2Y-A9H6 | 0.97808 | 0 | 0.6626 | 2.3379 | 13.758 | 7.4464 | 14.113 | 2.6541 | 0.469539101 | low |
| TCGA-K7-AAU7 | 0.98356 | 0 | 2.9841 | 5.7095 | 13.793 | 67.484 | 20.942 | 5.6063 | 0.954593761 | high |
| TCGA-5R-AA1C | 0.99726 | 0 | 5.4092 | 8.9379 | 9.1578 | 104.92 | 17.533 | 5.5522 | 1.153024343 | high |
| TCGA-CC-A7IH | 1 | 0 | 4.0797 | 2.4622 | 20.565 | 15.254 | 13.493 | 5.3197 | 0.618470955 | low |
| TCGA-DD-AACF | 1 | 1 | 6.1271 | 4.3709 | 10.662 | 10.081 | 34.701 | 7.9605 | 0.905746376 | high |
| TCGA-LG-A9QD | 1.00274 | 0 | 0.9679 | 1.7962 | 8.2426 | 6.4438 | 8.1977 | 2.5527 | 0.325882964 | low |
| TCGA-DD-A4NQ | 1.02192 | 1 | 4.3315 | 8.9255 | 16.642 | 73.288 | 30.479 | 6.953 | 1.293248821 | high |
| TCGA-LG-A6GG | 1.06027 | 0 | 1.7216 | 3.3354 | 10.512 | 23.749 | 17.02 | 3.5258 | 0.591391611 | low |
| TCGA-CC-A7II | 1.09315 | 0 | 11.219 | 9.0374 | 17.712 | 7.7416 | 55.699 | 9.4889 | 1.472451217 | high |
| TCGA-ED-A66X | 1.11233 | 0 | 3.5575 | 5.3442 | 17.976 | 11.289 | 31.564 | 5.3466 | 0.923978695 | high |
| TCGA-KR-A7K7 | 1.11507 | 0 | 5.4201 | 8.6239 | 9.8368 | 27.052 | 24.767 | 3.4988 | 0.955821887 | high |
| TCGA-ED-A82E | 1.11781 | 0 | 1.7964 | 6.228 | 25.885 | 9.8751 | 25.416 | 9.1781 | 1.038759133 | high |
| TCGA-G3-AAV3 | 1.12877 | 0 | 4.2941 | 3.4777 | 10.797 | 43.47 | 19.07 | 4.7771 | 0.715322042 | high |
| TCGA-YA-A8S7 | 1.12877 | 1 | 2.7044 | 7.1214 | 23.981 | 86.013 | 54.246 | 5.51 | 1.527990905 | high |
| TCGA-DD-AACP | 1.13699 | 0 | 5.4891 | 11.716 | 15.201 | 20.419 | 20.005 | 9.1221 | 1.202153716 | high |
| TCGA-DD-A1EL | 1.13699 | 1 | 8.7295 | 12.275 | 41.708 | 146.8 | 50.962 | 8.8662 | 2.169911857 | high |
| TCGA-G3-A25S | 1.13973 | 1 | 4.2975 | 7.967 | 11.473 | 39.292 | 25.053 | 4.8237 | 0.999085542 | high |
| TCGA-DD-A3A7 | 1.14795 | 1 | 2.2491 | 6.6714 | 7.6559 | 54.622 | 12.62 | 1.9458 | 0.742569525 | high |
| TCGA-BC-A217 | 1.15342 | 0 | 6.2146 | 2.2283 | 16.672 | 8.4766 | 28.155 | 6.8588 | 0.753003579 | high |
| TCGA-ED-A627 | 1.1589 | 0 | 1.4354 | 2.0417 | 10.444 | 10.658 | 18.099 | 4.069 | 0.510671225 | low |
| TCGA-LG-A9QC | 1.16438 | 0 | 3.2132 | 2.2255 | 15.171 | 29.943 | 13.139 | 2.6101 | 0.538741533 | low |
| TCGA-ED-A7XO | 1.16986 | 0 | 4.5822 | 2.1643 | 11.952 | 6.8678 | 15.104 | 4.3284 | 0.503129144 | low |
| TCGA-DD-AACQ | 1.18356 | 1 | 2.1875 | 4.7398 | 12.175 | 82.557 | 9.6262 | 2.5773 | 0.746405315 | high |
| TCGA-BC-A69H | 1.21644 | 0 | 7.2062 | 7.7376 | 17.469 | 14.738 | 17.977 | 4.2344 | 0.890500306 | high |
| TCGA-G3-A7M5 | 1.22466 | 0 | 2.0709 | 1.2157 | 7.4624 | 4.0833 | 13.936 | 2.0159 | 0.338937457 | low |
| TCGA-RC-A7SH | 1.28219 | 0 | 1.8517 | 5.1431 | 17.951 | 8.8911 | 24.397 | 4.2465 | 0.799623257 | high |
| TCGA-RC-A7SK | 1.29315 | 0 | 2.2126 | 3.5872 | 16.445 | 29.083 | 13.805 | 2.7341 | 0.621423644 | low |
| TCGA-DD-AAEB | 1.30959 | 0 | 1.068 | 1.9486 | 7.7994 | 4.7461 | 14.837 | 1.6111 | 0.378115738 | low |
| TCGA-G3-AAUZ | 1.31507 | 0 | 2.0496 | 3.2704 | 9.8841 | 5.5511 | 8.9895 | 2.0816 | 0.413905554 | low |
| TCGA-ED-A5KG | 1.32055 | 0 | 5.3226 | 3.7206 | 18.377 | 14.724 | 26.678 | 7.2114 | 0.848110449 | high |
| TCGA-BC-A3KG | 1.36438 | 0 | 12.854 | 4.8126 | 22.541 | 10.213 | 28.508 | 11.244 | 1.056640693 | high |
| TCGA-UB-A7MC | 1.36986 | 0 | 5.9988 | 4.9149 | 12.457 | 75.657 | 17.001 | 11.05 | 1.007347342 | high |
| TCGA-K7-A6G5 | 1.40274 | 0 | 1.1245 | 2.2571 | 10.97 | 19.776 | 13.964 | 3.0465 | 0.484804723 | low |
| TCGA-DD-A3A3 | 1.46575 | 1 | 1.9561 | 3.4912 | 6.0259 | 11.067 | 10.418 | 2.3114 | 0.427066907 | low |
| TCGA-BC-A5W4 | 1.49863 | 1 | 1.9772 | 4.9817 | 14.004 | 6.6983 | 21.583 | 4.2064 | 0.719672542 | high |
| TCGA-DD-AADU | 1.51781 | 0 | 1.2643 | 3.6518 | 5.6221 | 28.971 | 11.505 | 2.8321 | 0.50318531 | low |
| TCGA-DD-AAE0 | 1.52055 | 0 | 3.8742 | 12.099 | 18.167 | 5.3891 | 34.595 | 7.0975 | 1.315532491 | high |
| TCGA-2Y-A9H5 | 1.52055 | 1 | 2.3706 | 3.8374 | 10.423 | 9.0048 | 13.764 | 4.8478 | 0.567308985 | low |
| TCGA-DD-AADY | 1.52055 | 0 | 4.2064 | 4.8016 | 18.59 | 10.204 | 20.395 | 8.6677 | 0.848260112 | high |
| TCGA-WX-AA47 | 1.52329 | 1 | 0.258 | 3.3163 | 7.4218 | 6.9196 | 7.4614 | 2.1992 | 0.377850044 | low |
| TCGA-BC-A8YO | 1.53973 | 0 | 8.606 | 8.22 | 19.613 | 141.68 | 18.974 | 8.0534 | 1.39352007 | high |
| TCGA-DD-AAD1 | 1.54521 | 0 | 2.4585 | 2.9087 | 13.622 | 14.589 | 14.885 | 6.7822 | 0.617664236 | low |
| TCGA-DD-AAE3 | 1.55068 | 0 | 1.443 | 1.6875 | 9.3222 | 5.0565 | 12.106 | 1.9279 | 0.357398915 | low |
| TCGA-DD-AAEA | 1.57534 | 0 | 2.8966 | 4.2292 | 14.709 | 6.9906 | 20.134 | 2.6389 | 0.644282174 | low |
| TCGA-G3-A3CK | 1.60274 | 0 | 2.6816 | 2.3728 | 8.8639 | 69.356 | 13.188 | 3.2973 | 0.617182161 | low |
| TCGA-DD-A39Z | 1.64658 | 1 | 1.5054 | 2.985 | 12.894 | 2.2759 | 11.572 | 3.5644 | 0.473474279 | low |
| TCGA-DD-A1EC | 1.64932 | 0 | 2.1076 | 8.9204 | 9.91 | 11.821 | 17.032 | 5.4977 | 0.872244369 | high |
| TCGA-DD-A3A4 | 1.67671 | 1 | 0.9038 | 2.0428 | 16.459 | 21.291 | 12.386 | 3.1339 | 0.510234344 | low |
| TCGA-WX-AA44 | 1.68493 | 0 | 2.4161 | 9.1911 | 20.088 | 61.643 | 30.059 | 4.8753 | 1.24858192 | high |
| TCGA-G3-A5SL | 1.70137 | 0 | 3.4376 | 2.4703 | 11.475 | 19.217 | 11.938 | 3.9185 | 0.502516144 | low |
| TCGA-2Y-A9H8 | 1.73425 | 1 | 6.5552 | 1.5855 | 16.668 | 4.2052 | 18.38 | 3.8621 | 0.541161542 | low |
| TCGA-RC-A7S9 | 1.75342 | 0 | 2.2883 | 6.1537 | 14.025 | 4.6695 | 19.441 | 5.9833 | 0.786868128 | high |
| TCGA-DD-A4NE | 1.80822 | 1 | 6.248 | 5.6402 | 11.804 | 12.687 | 20.105 | 6.8746 | 0.805000213 | high |
| TCGA-G3-A3CG | 1.84384 | 0 | 1.4034 | 3.0516 | 10.525 | 17.389 | 24.475 | 3.7273 | 0.643493388 | low |
| TCGA-ES-A2HS | 1.88493 | 1 | 0.4905 | 1.6099 | 15.338 | 3.3539 | 10.126 | 1.5153 | 0.367606898 | low |
| TCGA-DD-A4NH | 1.89041 | 0 | 3.4873 | 6.9808 | 18.423 | 14.736 | 27.132 | 8.2581 | 1.030865432 | high |
| TCGA-DD-A73D | 1.89863 | 0 | 3.9245 | 3.625 | 18.995 | 29.41 | 7.9849 | 2.9298 | 0.593232181 | low |
| TCGA-XR-A8TF | 1.89863 | 1 | 5.4871 | 7.7774 | 20.664 | 26.285 | 17.678 | 5.6345 | 0.973088454 | high |
| TCGA-2Y-A9H9 | 1.90959 | 0 | 2.2725 | 1.8954 | 11.648 | 16.093 | 13.01 | 3.7487 | 0.470390628 | low |
| TCGA-G3-A5SJ | 1.91233 | 0 | 4.0464 | 3.1237 | 15.842 | 15.39 | 20.442 | 4.6138 | 0.671691765 | low |
| TCGA-DD-A73C | 1.92055 | 0 | 2.2518 | 2.1625 | 16.383 | 18.454 | 12.967 | 3.6195 | 0.528648879 | low |
| TCGA-ZP-A9D0 | 1.96438 | 0 | 0.7382 | 1.9021 | 13.759 | 11.847 | 6.4592 | 1.8492 | 0.360552572 | low |
| TCGA-DD-AAEG | 1.96986 | 0 | 7.0027 | 4.3323 | 11.467 | 34.095 | 11.247 | 5.1693 | 0.669257919 | low |
| TCGA-2Y-A9GS | 1.98356 | 1 | 8.7029 | 5.6042 | 19.328 | 74.292 | 31.889 | 7.3627 | 1.193948139 | high |
| TCGA-ZP-A9D2 | 2.03562 | 0 | 3.4968 | 3.5425 | 17.704 | 40.284 | 28.372 | 8.335 | 0.942031745 | high |
| TCGA-MI-A75H | 2.04658 | 0 | 2.6074 | 3.3895 | 13.266 | 30.809 | 20.259 | 3.6109 | 0.678814943 | low |
| TCGA-HP-A5N0 | 2.06027 | 1 | 2.0336 | 2.4686 | 10.822 | 13.375 | 12.253 | 2.826 | 0.455662433 | low |
| TCGA-BC-A10X | 2.10959 | 1 | 1.344 | 1.2935 | 8.0174 | 9.2644 | 13.66 | 3.2001 | 0.381408104 | low |
| TCGA-ZP-A9CY | 2.14247 | 0 | 1.6324 | 1.9294 | 9.144 | 15.641 | 12.624 | 2.9102 | 0.42500458 | low |
| TCGA-DD-AAEH | 2.14795 | 0 | 1.0349 | 2.2121 | 15.91 | 25.28 | 15.005 | 2.9988 | 0.551636236 | low |
| TCGA-DD-A4NG | 2.19726 | 1 | 2.1067 | 5.0681 | 13.02 | 85.922 | 20.236 | 4.6406 | 0.938067828 | high |
| TCGA-DD-AAEE | 2.21918 | 0 | 3.685 | 3.4529 | 11.928 | 23.626 | 17.659 | 4.1641 | 0.636571919 | low |
| TCGA-DD-A39W | 2.26575 | 1 | 3.5932 | 5.0117 | 11.252 | 15.423 | 11.9 | 6.9318 | 0.67869815 | low |
| TCGA-BC-A10U | 2.29315 | 1 | 1.9845 | 3.4989 | 15.401 | 42.863 | 20.582 | 3.156 | 0.729464374 | high |
| TCGA-G3-A25V | 2.35616 | 0 | 0.7113 | 2.9439 | 8.1612 | 59.538 | 13.829 | 2.4808 | 0.594184523 | low |
| TCGA-DD-AADN | 2.46027 | 0 | 1.7122 | 5.3491 | 9.3237 | 25.299 | 22.854 | 2.9017 | 0.736515069 | high |
| TCGA-XR-A8TE | 2.53425 | 0 | 1.1863 | 5.5188 | 9.844 | 16.121 | 14.205 | 4.4357 | 0.658457424 | low |
| TCGA-DD-A3A9 | 2.55068 | 1 | 0.8915 | 5.5375 | 15.378 | 4.6638 | 22.289 | 4.7045 | 0.767565814 | high |
| TCGA-DD-AADK | 2.87397 | 0 | 2.2519 | 5.2428 | 7.7528 | 7.923 | 19.307 | 4.6582 | 0.667039321 | low |
| TCGA-DD-AADJ | 2.92055 | 0 | 1.8222 | 4.2403 | 10.227 | 13.894 | 20.519 | 5.3509 | 0.681930491 | low |
| TCGA-DD-AADI | 2.9726 | 0 | 4.1965 | 2.6883 | 12.936 | 4.2101 | 20.411 | 7.8215 | 0.658985376 | low |
| TCGA-ZP-A9CV | 2.98082 | 1 | 2.5407 | 3.7781 | 9.6386 | 47.953 | 11.105 | 3.9571 | 0.622735824 | low |
| TCGA-BD-A3ER | 3.05479 | 0 | 0.7222 | 5.4462 | 8.053 | 37.74 | 14.211 | 3.2291 | 0.674827465 | low |
| TCGA-2Y-A9H7 | 3.2 | 0 | 2.1126 | 5.9924 | 15.924 | 6.0304 | 15.186 | 5.2206 | 0.736465941 | high |
| TCGA-DD-AAD8 | 3.33973 | 0 | 1.8896 | 4.2764 | 9.166 | 32.222 | 18.02 | 3.1098 | 0.653783841 | low |
| TCGA-DD-AADA | 3.37808 | 0 | 0.8108 | 1.395 | 6.7792 | 5.3313 | 12.02 | 3.2222 | 0.34492912 | low |
| TCGA-ZS-A9CE | 3.4 | 0 | 4.0513 | 1.8737 | 13.507 | 10.72 | 13.071 | 2.0337 | 0.441742892 | low |
| TCGA-DD-AAD3 | 3.54795 | 0 | 1.5961 | 1.6104 | 10.174 | 14.771 | 16.546 | 3.3077 | 0.466545429 | low |
| TCGA-DD-AAD5 | 3.68493 | 0 | 2.1771 | 13.961 | 19.453 | 56.876 | 27.292 | 4.8501 | 1.435245176 | high |
| TCGA-ZS-A9CD | 3.79726 | 1 | 1.2818 | 4.1509 | 10.256 | 44.226 | 16.007 | 2.7434 | 0.659839825 | low |
| TCGA-BC-A10S | 3.89863 | 1 | 1.1672 | 3.3066 | 8.3721 | 6.8488 | 16.216 | 2.6258 | 0.493213451 | low |
| TCGA-DD-AACY | 3.9726 | 0 | 1.5897 | 2.3493 | 10.003 | 14.354 | 7.5745 | 3.0225 | 0.396741099 | low |
| TCGA-2Y-A9H4 | 3.97808 | 0 | 4.0772 | 2.0842 | 13.574 | 9.0949 | 14.51 | 3.9301 | 0.503170183 | low |
| TCGA-BC-4072 | 4.08219 | 1 | 2.2824 | 3.6186 | 16.127 | 15.337 | 17.662 | 3.5455 | 0.639814407 | low |
| TCGA-DD-A1EH | 4.09589 | 0 | 3.5997 | 3.1449 | 16.999 | 10.267 | 19.446 | 8.5229 | 0.736472849 | high |
| TCGA-DD-AAEI | 4.19452 | 0 | 2.6347 | 3.5312 | 11.984 | 108.36 | 12.354 | 2.8243 | 0.795321075 | high |
| TCGA-DD-AACT | 4.27945 | 0 | 1.539 | 2.1883 | 7.2116 | 30.524 | 11.481 | 2.4016 | 0.440491973 | low |
| TCGA-DD-AACI | 4.43288 | 0 | 2.1663 | 3.7321 | 4.6611 | 40.648 | 21.759 | 2.5097 | 0.640980816 | low |
| TCGA-DD-A116 | 4.44384 | 1 | 2.7648 | 3.5614 | 12.392 | 12.452 | 22.757 | 5.904 | 0.702195968 | high |
| TCGA-G3-A25U | 4.48219 | 0 | 6.02 | 3.5087 | 11.232 | 13.93 | 6.0727 | 5.71 | 0.519261741 | low |
| TCGA-DD-A4NL | 4.68767 | 0 | 0.7118 | 0.8263 | 6.8355 | 6.9292 | 8.0104 | 1.7897 | 0.247920117 | low |
| TCGA-2Y-A9H2 | 4.74247 | 0 | 2.1904 | 9.8797 | 14.307 | 15.202 | 11.09 | 5.3144 | 0.899797343 | high |
| TCGA-G3-A25X | 4.87397 | 0 | 4.9825 | 8.8277 | 15.853 | 6.8521 | 26.559 | 9.5331 | 1.103226182 | high |
| TCGA-NI-A4U2 | 4.90685 | 1 | 0.8691 | 1.7552 | 10.061 | 9.9422 | 12.794 | 2.8826 | 0.40646876 | low |
| TCGA-DD-AACS | 4.94247 | 0 | 6.8068 | 2.6987 | 11.562 | 32.01 | 12.232 | 12.141 | 0.736968152 | high |
| TCGA-DD-AAVS | 4.99452 | 0 | 2.9583 | 6.1601 | 15.383 | 16.158 | 22.932 | 8.0686 | 0.915797182 | high |
| TCGA-FV-A23B | 5.07397 | 1 | 1.9328 | 6.5102 | 17.241 | 9.2053 | 13.514 | 4.4863 | 0.748794453 | high |
| TCGA-DD-AAW2 | 5.08219 | 0 | 3.3305 | 2.1852 | 8.4982 | 15.182 | 16.939 | 4.1251 | 0.509195953 | low |
| TCGA-DD-AACO | 5.13973 | 0 | 4.7926 | 3.1247 | 10.486 | 23.835 | 24.954 | 2.0085 | 0.647325136 | low |
| TCGA-2Y-A9GU | 5.31233 | 0 | 1.182 | 2.1081 | 12.806 | 7.477 | 10.351 | 3.7333 | 0.432987486 | low |
| TCGA-DD-AAW1 | 5.44932 | 0 | 2.0177 | 1.8701 | 6.9425 | 21.005 | 8.0149 | 2.8651 | 0.368372065 | low |
| TCGA-DD-AAW0 | 5.52055 | 0 | 1.5202 | 2.9283 | 8.5313 | 20.844 | 16.364 | 5.0335 | 0.568726292 | low |
| TCGA-DD-A4NV | 5.52877 | 0 | 1.3437 | 2.9005 | 9.9706 | 10.539 | 11.109 | 4.9197 | 0.489887183 | low |
| TCGA-BC-A110 | 5.79726 | 1 | 0.7701 | 2.0614 | 6.9353 | 8.2506 | 14.404 | 2.6791 | 0.402543012 | low |
| TCGA-DD-A3A2 | 5.83836 | 1 | 1.1702 | 1.4866 | 14.269 | 7.972 | 11.497 | 2.187 | 0.396744976 | low |
| TCGA-DD-AACE | 5.98356 | 0 | 1.3707 | 2.1676 | 8.7151 | 9.8596 | 14.128 | 3.2031 | 0.438021973 | low |
| TCGA-DD-A4ND | 6.11507 | 0 | 3.9684 | 2.6629 | 14.016 | 7.7264 | 13.173 | 4.5776 | 0.530299768 | low |
| TCGA-DD-A4NO | 6.15068 | 0 | 1.3559 | 6.7594 | 9.4537 | 38.884 | 24.047 | 4.1368 | 0.884252824 | high |
| TCGA-DD-A1ED | 6.30411 | 0 | 1.0728 | 1.0915 | 8.7124 | 8.28 | 9.3212 | 2.2724 | 0.307034348 | low |
| TCGA-DD-AACA | 6.30411 | 0 | 5.1345 | 2.9179 | 10.965 | 13.115 | 19.12 | 7.1402 | 0.654028458 | low |
| TCGA-DD-AAVW | 6.34795 | 0 | 1.8502 | 2.2533 | 11.465 | 21.728 | 17.883 | 4.222 | 0.564307545 | low |
| TCGA-ZS-A9CF | 6.60822 | 0 | 3.6922 | 3.586 | 8.2166 | 10.703 | 9.1172 | 5.1447 | 0.500581655 | low |
| TCGA-DD-A1EA | 6.61644 | 0 | 3.6706 | 2.7627 | 12.726 | 48.546 | 18.756 | 6.29 | 0.736771889 | high |
| TCGA-DD-A113 | 6.64384 | 0 | 5.8222 | 7.5246 | 17.742 | 47.327 | 13.211 | 6.9636 | 0.975429853 | high |
| TCGA-2Y-A9GX | 6.69041 | 0 | 1.9881 | 4.4349 | 8.559 | 50.996 | 22.735 | 4.3265 | 0.787420408 | high |
| TCGA-DD-AAVV | 6.72603 | 0 | 2.8091 | 5.5347 | 11.849 | 42.115 | 17.228 | 4.6357 | 0.79460982 | high |
| TCGA-DD-A4NS | 6.72877 | 1 | 1.8214 | 2.12 | 11.427 | 17.569 | 16.979 | 3.9811 | 0.530402291 | low |
| TCGA-DD-AAVR | 6.88493 | 0 | 1.1857 | 2.1231 | 10.768 | 15.579 | 18.988 | 2.7983 | 0.51424984 | low |
| TCGA-DD-A115 | 6.96438 | 1 | 8.1257 | 2.7794 | 12.344 | 16.661 | 14.243 | 5.0824 | 0.584410231 | low |
| TCGA-DD-AAVQ | 7.47397 | 0 | 4.4166 | 4.2581 | 12.386 | 5.2475 | 9.8494 | 5.2662 | 0.567936101 | low |
| TCGA-DD-A3A6 | 8.92603 | 1 | 0.1316 | 1.1284 | 8.7379 | 8.1467 | 11.742 | 3.4293 | 0.355663615 | low |
| TCGA-DD-A73G | 9.52877 | 0 | 0.7152 | 2.483 | 18.567 | 3.5873 | 13.735 | 2.2959 | 0.496367028 | low |

| Validation cohort | |  |  |  |  |  |  |  |  |  |
| --- | --- | --- | --- | --- | --- | --- | --- | --- | --- | --- |
| Id | futime | fustat | PRIM1 | UCK2 | SEPHS1 | TXNRD1 | SMS | GMPS | riskScore | risk |
| TCGA-CC-A9FV | 0.002739726 | 0 | 1.020927 | 0.8890442 | 14.12343 | 9.081439 | 27.0976 | 5.877643 | 0.61508065 | low |
| TCGA-CC-A9FU | 0.002739726 | 0 | 4.296176 | 10.46375 | 39.21654 | 13.77338 | 2.746429 | 5.154605 | 1.055716145 | high |
| TCGA-2V-A95S | 0.002739726 | 0 | 5.439543 | 5.035154 | 9.920127 | 53.29377 | 20.55985 | 4.253494 | 0.823076157 | high |
| TCGA-BW-A5NQ | 0.002739726 | 0 | 13.74453 | 6.049362 | 12.24776 | 68.2115 | 42.74625 | 11.11864 | 1.351295911 | high |
| TCGA-FV-A495 | 0.002739726 | 0 | 4.737777 | 3.909116 | 13.64881 | 5.628231 | 15.34064 | 3.065785 | 0.578318368 | low |
| TCGA-ED-A7PZ | 0.016438356 | 0 | 4.278381 | 3.685721 | 11.54705 | 17.53906 | 13.54817 | 5.34478 | 0.608990047 | low |
| TCGA-ED-A7PX | 0.016438356 | 0 | 1.085192 | 5.517693 | 21.1039 | 31.63922 | 41.94756 | 7.795819 | 1.173605759 | high |
| TCGA-DD-AACK | 0.024657534 | 0 | 2.747273 | 4.337432 | 9.278419 | 172.7677 | 13.74954 | 4.736061 | 1.052343606 | high |
| TCGA-DD-A4NR | 0.024657534 | 1 | 4.553504 | 6.683372 | 15.4025 | 58.955 | 22.12646 | 4.214118 | 0.982448981 | high |
| TCGA-RC-A6M6 | 0.024657534 | 0 | 4.213857 | 8.670163 | 16.74023 | 101.1295 | 41.7058 | 5.088702 | 1.445135901 | high |
| TCGA-FV-A496 | 0.02739726 | 0 | 3.081431 | 5.246184 | 9.089657 | 14.7266 | 5.329725 | 2.706895 | 0.508466119 | low |
| TCGA-DD-A3A8 | 0.030136986 | 1 | 3.282153 | 2.146047 | 10.87192 | 7.551856 | 12.91115 | 3.329344 | 0.445416091 | low |
| TCGA-FV-A4ZQ | 0.032876712 | 0 | 7.321679 | 5.665765 | 21.45776 | 11.23588 | 24.41905 | 6.804841 | 0.936056025 | high |
| TCGA-RC-A6M5 | 0.04109589 | 0 | 0.5991182 | 1.157244 | 11.31024 | 12.81843 | 9.848512 | 2.49672 | 0.3547539 | low |
| TCGA-EP-A2KC | 0.052054795 | 1 | 2.296556 | 5.731529 | 11.82624 | 36.30131 | 18.37229 | 3.618017 | 0.777080461 | high |
| TCGA-5C-AAPD | 0.054794521 | 0 | 9.927291 | 5.256139 | 19.58864 | 7.309863 | 60.42434 | 11.12233 | 1.380010114 | high |
| TCGA-T1-A6J8 | 0.063013699 | 0 | 1.618561 | 3.766041 | 13.0059 | 46.22573 | 23.43529 | 3.337571 | 0.765103335 | high |
| TCGA-G3-AAV4 | 0.073972603 | 1 | 7.071792 | 3.146695 | 14.20813 | 26.33998 | 27.17037 | 5.589891 | 0.794712913 | high |
| TCGA-GJ-A6C0 | 0.084931507 | 1 | 3.258529 | 5.584468 | 14.69752 | 21.88755 | 30.302 | 4.592811 | 0.907321899 | high |
| TCGA-2Y-A9HA | 0.098630137 | 1 | 5.902543 | 1.711978 | 16.99731 | 58.40105 | 21.86081 | 4.577821 | 0.756736399 | high |
| TCGA-DD-A73E | 0.120547945 | 0 | 4.30497 | 2.10411 | 11.8681 | 35.54512 | 10.48154 | 6.251478 | 0.570203124 | low |
| TCGA-5R-AAAM | 0.126027397 | 1 | 1.605162 | 1.767146 | 9.712218 | 28.86093 | 13.74482 | 3.094599 | 0.475892003 | low |
| TCGA-G3-A7M9 | 0.153424658 | 1 | 11.9678 | 10.41638 | 28.76313 | 19.75482 | 49.92635 | 12.88253 | 1.682151369 | high |
| TCGA-ED-A8O6 | 0.153424658 | 1 | 3.198454 | 6.830667 | 18.27501 | 17.33466 | 25.43429 | 4.033374 | 0.922600057 | high |
| TCGA-GJ-A9DB | 0.183561644 | 1 | 3.860225 | 2.769034 | 11.94363 | 17.74257 | 17.47066 | 4.186932 | 0.584765017 | low |
| TCGA-HP-A5MZ | 0.249315068 | 1 | 1.340309 | 3.455319 | 9.938886 | 47.74676 | 27.11693 | 2.814292 | 0.75557387 | high |
| TCGA-DD-AACL | 0.293150685 | 1 | 8.700664 | 11.28385 | 27.03448 | 15.03594 | 45.37718 | 13.47364 | 1.647349935 | high |
| TCGA-CC-5263 | 0.353424658 | 1 | 8.080395 | 3.094949 | 21.16525 | 9.157679 | 23.62207 | 13.79302 | 0.938081917 | high |
| TCGA-DD-AAD0 | 0.375342466 | 0 | 4.440435 | 2.973305 | 9.023701 | 22.28629 | 12.67263 | 5.85711 | 0.56673827 | low |
| TCGA-DD-AAE6 | 0.38630137 | 0 | 3.111846 | 6.24706 | 18.97865 | 14.25041 | 6.144094 | 4.112437 | 0.681454521 | low |
| TCGA-BC-A112 | 0.419178082 | 1 | 3.860661 | 9.409317 | 12.01366 | 83.08667 | 20.85366 | 5.610826 | 1.170492882 | high |
| TCGA-DD-AACX | 0.465753425 | 0 | 2.632397 | 4.25471 | 10.3706 | 99.49038 | 20.98023 | 4.012768 | 0.910506015 | high |
| TCGA-DD-A39Y | 0.468493151 | 1 | 3.109685 | 11.70445 | 13.81981 | 76.96129 | 23.79004 | 5.877892 | 1.31782049 | high |
| TCGA-G3-A3CI | 0.493150685 | 0 | 0.6888581 | 0.9724558 | 9.435117 | 4.081408 | 10.2475 | 2.162333 | 0.301786965 | low |
| TCGA-DD-A1EI | 0.501369863 | 0 | 5.276113 | 5.218534 | 17.42137 | 28.79214 | 22.93801 | 7.422405 | 0.918196571 | high |
| TCGA-CC-A7IE | 0.594520548 | 1 | 5.930626 | 9.069559 | 12.80144 | 16.77595 | 38.03857 | 3.854834 | 1.128524027 | high |
| TCGA-DD-A3A1 | 0.638356164 | 1 | 1.767864 | 6.921426 | 7.646922 | 121.251 | 18.27107 | 3.309632 | 1.034849176 | high |
| TCGA-CC-A9FW | 0.679452055 | 0 | 6.259187 | 5.117987 | 15.70581 | 33.49263 | 27.74197 | 4.284516 | 0.902850566 | high |
| TCGA-CC-5259 | 0.684931507 | 0 | 0.9046691 | 3.878653 | 11.89179 | 4.681116 | 32.69107 | 2.552245 | 0.723680638 | high |
| TCGA-2Y-A9HB | 0.712328767 | 0 | 1.968712 | 3.750963 | 10.63272 | 15.46345 | 11.74888 | 4.019425 | 0.542829504 | low |
| TCGA-CC-A7IL | 0.761643836 | 1 | 1.995217 | 4.509142 | 11.62908 | 13.60654 | 13.32121 | 3.997616 | 0.600769902 | low |
| TCGA-CC-A8HV | 0.764383562 | 1 | 3.368292 | 11.62229 | 20.36532 | 10.91481 | 10.89132 | 6.818757 | 1.06041701 | high |
| TCGA-CC-A3M9 | 0.821917808 | 1 | 4.545513 | 11.0408 | 13.00959 | 10.07974 | 122.5154 | 9.109454 | 2.235516969 | high |
| TCGA-CC-A3MA | 0.830136986 | 1 | 2.287908 | 16.86101 | 37.94673 | 31.58809 | 33.54829 | 11.36792 | 1.872092173 | high |
| TCGA-CC-A5UD | 0.832876712 | 1 | 6.568069 | 11.22858 | 39.80417 | 87.95683 | 18.12705 | 7.550507 | 1.538185681 | high |
| TCGA-BC-A10R | 0.843835616 | 1 | 0.4716095 | 1.757132 | 31.20967 | 21.53405 | 30.23806 | 4.472933 | 0.846744666 | high |
| TCGA-CC-A3MB | 0.863013699 | 1 | 4.182226 | 8.651478 | 35.70451 | 37.57744 | 31.73743 | 6.598384 | 1.349338695 | high |
| TCGA-5C-A9VG | 0.898630137 | 0 | 2.71581 | 17.32121 | 21.3103 | 16.21493 | 19.45695 | 6.636585 | 1.455148396 | high |
| TCGA-EP-A12J | 0.904109589 | 0 | 4.563803 | 1.870318 | 9.822583 | 25.05383 | 9.391676 | 4.016573 | 0.453301977 | low |
| TCGA-5R-AA1D | 0.923287671 | 0 | 0.8329561 | 2.011249 | 11.41688 | 11.63199 | 11.3732 | 3.312747 | 0.429180186 | low |
| TCGA-ZS-A9CG | 0.934246575 | 0 | 2.528311 | 2.663292 | 7.381443 | 9.908519 | 10.15922 | 3.379809 | 0.415699382 | low |
| TCGA-WJ-A86L | 0.945205479 | 0 | 1.932248 | 4.693971 | 15.29128 | 18.49437 | 13.26622 | 3.880673 | 0.652802414 | low |
| TCGA-DD-AAC9 | 0.950684932 | 0 | 1.722254 | 3.155927 | 6.56342 | 14.64832 | 13.29092 | 2.415908 | 0.458062428 | low |
| TCGA-DD-A1EE | 0.956164384 | 1 | 3.056951 | 11.94885 | 11.89708 | 213.6279 | 17.61946 | 6.068585 | 1.642808305 | high |
| TCGA-EP-A2KA | 0.978082192 | 0 | 4.002512 | 3.384703 | 16.29178 | 11.01325 | 17.61554 | 3.936875 | 0.630994862 | low |
| TCGA-G3-AAV1 | 0.983561644 | 1 | 5.512004 | 5.254714 | 8.418473 | 91.62641 | 16.61158 | 4.191196 | 0.887041002 | high |
| TCGA-G3-AAV7 | 0.989041096 | 0 | 8.712583 | 8.902381 | 32.27035 | 32.72814 | 43.01079 | 7.188635 | 1.46963145 | high |
| TCGA-G3-A7M7 | 0.989041096 | 0 | 0.5960132 | 3.294432 | 13.61374 | 36.46304 | 12.59848 | 2.644465 | 0.582437051 | low |
| TCGA-EP-A3RK | 0.994520548 | 0 | 3.82705 | 7.106697 | 12.94741 | 68.833 | 32.93603 | 5.794393 | 1.158601795 | high |
| TCGA-CC-A3MC | 0.994520548 | 0 | 4.581857 | 17.05946 | 8.981514 | 48.97467 | 20.1439 | 8.048652 | 1.472095658 | high |
| TCGA-FV-A3R3 | 1.002739726 | 1 | 0.5300732 | 1.22425 | 16.33563 | 11.76576 | 13.46444 | 2.806831 | 0.444643388 | low |
| TCGA-G3-AAV2 | 1.019178082 | 0 | 2.297617 | 3.084934 | 8.41086 | 8.805122 | 16.55269 | 3.586429 | 0.515783139 | low |
| TCGA-DD-AACD | 1.043835616 | 1 | 0.7859347 | 2.00244 | 10.20792 | 5.732248 | 16.43245 | 2.26483 | 0.434606837 | low |
| TCGA-CC-A7IJ | 1.046575342 | 0 | 4.55122 | 5.391491 | 16.21319 | 52.00574 | 89.8936 | 13.33027 | 1.833869826 | high |
| TCGA-ED-A4XI | 1.057534247 | 0 | 1.230929 | 1.817178 | 9.40144 | 19.69583 | 11.47033 | 2.996918 | 0.421075015 | low |
| TCGA-BC-A69I | 1.060273973 | 0 | 1.404148 | 2.882221 | 10.02709 | 7.248004 | 10.5143 | 2.748659 | 0.428816196 | low |
| TCGA-ED-A7PY | 1.068493151 | 0 | 2.307622 | 4.268942 | 14.10056 | 4.758476 | 20.27572 | 5.017556 | 0.683133854 | low |
| TCGA-DD-A4NB | 1.071232877 | 0 | 0.8646375 | 2.244377 | 13.20352 | 34.77193 | 14.18329 | 3.910732 | 0.566166633 | low |
| TCGA-DD-A1EF | 1.079452055 | 1 | 7.446316 | 10.49396 | 21.24451 | 8.572776 | 20.35127 | 7.500517 | 1.137387643 | high |
| TCGA-ZP-A9D4 | 1.082191781 | 0 | 3.928963 | 2.848242 | 11.8591 | 69.71725 | 10.11371 | 3.760251 | 0.648604468 | low |
| TCGA-WQ-AB4B | 1.082191781 | 0 | 1.029867 | 2.824219 | 10.73276 | 13.55498 | 19.31897 | 1.980725 | 0.52916316 | low |
| TCGA-3K-AAZ8 | 1.084931507 | 0 | 2.193322 | 3.016839 | 11.12664 | 12.76856 | 13.56318 | 5.37684 | 0.551538983 | low |
| TCGA-ED-A7XP | 1.095890411 | 0 | 4.338001 | 5.867224 | 9.925843 | 79.09678 | 24.00499 | 6.207172 | 1.012850326 | high |
| TCGA-ED-A8O5 | 1.112328767 | 0 | 4.310425 | 1.337947 | 14.99826 | 7.227134 | 15.45597 | 6.040526 | 0.527907784 | low |
| TCGA-ED-A459 | 1.117808219 | 0 | 4.240255 | 5.418587 | 16.19276 | 36.15781 | 67.38447 | 7.056264 | 1.412706435 | high |
| TCGA-BD-A3EP | 1.120547945 | 0 | 1.859375 | 3.732782 | 12.46001 | 40.94063 | 18.73762 | 4.640678 | 0.71998839 | high |
| TCGA-DD-AA3A | 1.123287671 | 1 | 2.98443 | 14.42132 | 25.7415 | 26.99961 | 34.52986 | 12.17914 | 1.660733799 | high |
| TCGA-DD-AADC | 1.164383562 | 1 | 16.67735 | 7.740129 | 19.61059 | 46.77796 | 25.83043 | 6.787799 | 1.174496386 | high |
| TCGA-DD-A4NF | 1.17260274 | 0 | 2.821704 | 5.147575 | 15.34276 | 31.19608 | 18.90131 | 4.628862 | 0.792637887 | high |
| TCGA-G3-A7M8 | 1.178082192 | 0 | 1.420514 | 2.261933 | 10.33036 | 5.092265 | 11.4667 | 2.462585 | 0.39891756 | low |
| TCGA-DD-AADQ | 1.194520548 | 0 | 0.9742465 | 1.934375 | 8.995259 | 11.4123 | 14.92979 | 2.793017 | 0.432158703 | low |
| TCGA-ES-A2HT | 1.2 | 1 | 0.4109547 | 1.564736 | 7.797105 | 6.218416 | 18.39168 | 1.773067 | 0.403001143 | low |
| TCGA-G3-A25Y | 1.238356164 | 1 | 5.559378 | 8.386424 | 18.07174 | 9.960693 | 28.85136 | 10.4342 | 1.155359405 | high |
| TCGA-DD-AADO | 1.24109589 | 0 | 3.473759 | 3.82787 | 8.50308 | 4.125916 | 10.29166 | 2.271492 | 0.448812952 | low |
| TCGA-DD-AADP | 1.254794521 | 0 | 3.13775 | 4.435082 | 16.75473 | 10.9595 | 16.46934 | 5.834978 | 0.710799054 | high |
| TCGA-DD-AACG | 1.284931507 | 1 | 6.917514 | 5.49038 | 10.33191 | 10.83604 | 25.22948 | 5.235346 | 0.803842927 | high |
| TCGA-DD-AADS | 1.298630137 | 0 | 0.6523526 | 1.543045 | 9.759852 | 3.216958 | 18.97943 | 0.6717167 | 0.394955573 | low |
| TCGA-G3-AAV0 | 1.304109589 | 0 | 0.8005785 | 2.229664 | 7.993837 | 3.175656 | 7.288979 | 2.277553 | 0.31966839 | low |
| TCGA-UB-A7ME | 1.331506849 | 0 | 11.43746 | 3.845293 | 12.34275 | 5.962511 | 20.19571 | 4.345231 | 0.668484296 | low |
| TCGA-MI-A75E | 1.389041096 | 0 | 1.337167 | 3.786632 | 10.04319 | 33.96619 | 17.09799 | 2.740173 | 0.622364349 | low |
| TCGA-K7-A5RG | 1.421917808 | 0 | 7.147328 | 2.337851 | 8.834625 | 68.65276 | 21.29258 | 4.792637 | 0.748809054 | high |
| TCGA-G3-A5SM | 1.424657534 | 0 | 1.739475 | 3.210981 | 14.28371 | 42.9512 | 23.4386 | 3.342955 | 0.739794312 | high |
| TCGA-UB-A7MA | 1.465753425 | 0 | 8.417583 | 8.539764 | 25.13235 | 17.6352 | 22.8947 | 6.088934 | 1.102235402 | high |
| TCGA-O8-A75V | 1.473972603 | 0 | 1.814851 | 4.23695 | 9.129995 | 22.49813 | 15.4749 | 2.957083 | 0.59230145 | low |
| TCGA-DD-AAE1 | 1.512328767 | 0 | 3.109839 | 11.35739 | 12.32072 | 7.887262 | 17.25195 | 9.678846 | 1.095882721 | high |
| TCGA-DD-A1EK | 1.528767123 | 1 | 1.037579 | 3.700743 | 10.898 | 13.36235 | 19.26631 | 3.492209 | 0.604419795 | low |
| TCGA-DD-A4NI | 1.536986301 | 0 | 1.332792 | 1.300238 | 7.055289 | 13.22087 | 10.02018 | 3.085673 | 0.342559535 | low |
| TCGA-DD-AADV | 1.57260274 | 0 | 8.350247 | 2.707907 | 15.29073 | 36.45668 | 16.15327 | 5.181342 | 0.68730049 | low |
| TCGA-RC-A7SF | 1.58630137 | 0 | 1.529577 | 3.28662 | 6.687503 | 24.72141 | 13.86976 | 3.042932 | 0.513232931 | low |
| TCGA-FV-A2QR | 1.591780822 | 1 | 2.416308 | 4.45788 | 10.59654 | 18.90706 | 15.74178 | 2.798282 | 0.607601893 | low |
| TCGA-DD-AADW | 1.608219178 | 0 | 5.053439 | 7.046988 | 26.90552 | 16.75759 | 26.49047 | 11.25142 | 1.174711919 | high |
| TCGA-RC-A7SB | 1.610958904 | 0 | 2.415223 | 1.915673 | 14.48061 | 11.45165 | 26.66119 | 3.329204 | 0.623731381 | low |
| TCGA-G3-A3CJ | 1.62739726 | 0 | 1.104093 | 3.169521 | 7.151142 | 14.86908 | 11.28035 | 2.027301 | 0.432272568 | low |
| TCGA-UB-A7MB | 1.646575342 | 0 | 9.752927 | 14.83963 | 18.54384 | 15.62287 | 13.21139 | 8.52994 | 1.302165727 | high |
| TCGA-NI-A8LF | 1.660273973 | 0 | 0.7801947 | 1.759565 | 6.61287 | 15.42338 | 15.09902 | 2.190062 | 0.402843759 | low |
| TCGA-DD-AAE4 | 1.665753425 | 0 | 3.619803 | 1.729979 | 9.827102 | 3.729476 | 15.47285 | 2.336483 | 0.413231194 | low |
| TCGA-MI-A75I | 1.726027397 | 0 | 2.369273 | 2.662074 | 15.42758 | 33.7205 | 19.02785 | 4.898025 | 0.682173159 | low |
| TCGA-K7-A5RF | 1.728767123 | 0 | 0.9064573 | 0.9283727 | 7.852891 | 8.150515 | 11.96196 | 2.128855 | 0.316292147 | low |
| TCGA-G3-A7M6 | 1.731506849 | 0 | 2.801071 | 9.609592 | 23.33399 | 11.88867 | 18.29906 | 5.169401 | 1.033667068 | high |
| TCGA-DD-AADL | 1.742465753 | 0 | 5.811592 | 4.389697 | 13.88155 | 22.13729 | 22.1274 | 6.487933 | 0.800591405 | high |
| TCGA-DD-AAE2 | 1.747945205 | 0 | 4.945563 | 1.781509 | 7.3601 | 13.01879 | 10.4964 | 3.700674 | 0.399640311 | low |
| TCGA-PD-A5DF | 1.750684932 | 1 | 4.061952 | 3.861158 | 11.8548 | 18.37351 | 20.791 | 5.694403 | 0.70836687 | high |
| TCGA-DD-A39V | 1.761643836 | 1 | 4.216034 | 7.27992 | 8.791643 | 45.66839 | 19.03832 | 2.98196 | 0.855702233 | high |
| TCGA-DD-AAE7 | 1.764383562 | 0 | 1.785991 | 1.170911 | 7.705481 | 5.939075 | 11.59009 | 2.174005 | 0.32078248 | low |
| TCGA-CC-A7IF | 1.778082192 | 1 | 3.356438 | 4.356167 | 15.35046 | 11.66382 | 9.971457 | 3.529785 | 0.578768276 | low |
| TCGA-G3-A25Z | 1.794520548 | 0 | 3.610795 | 3.843046 | 10.13055 | 13.48964 | 15.93297 | 2.74271 | 0.562594158 | low |
| TCGA-KR-A7K2 | 1.8 | 0 | 1.448642 | 1.850694 | 11.2177 | 4.502686 | 12.55323 | 2.530265 | 0.397866017 | low |
| TCGA-DD-AAD2 | 1.802739726 | 0 | 2.118388 | 3.130247 | 8.485828 | 18.97838 | 14.17046 | 3.058554 | 0.510382606 | low |
| TCGA-DD-A11C | 1.81369863 | 0 | 3.789743 | 6.4807 | 14.39911 | 9.544145 | 11.57354 | 5.449146 | 0.728923496 | high |
| TCGA-G3-A6UC | 1.838356164 | 0 | 3.057735 | 2.627343 | 10.36433 | 11.97743 | 8.571577 | 3.027861 | 0.423237697 | low |
| TCGA-DD-AAD6 | 1.84109589 | 0 | 3.85182 | 4.145278 | 7.213037 | 51.82246 | 17.38312 | 3.47069 | 0.694217076 | high |
| TCGA-MI-A75G | 1.912328767 | 0 | 1.639194 | 4.006144 | 12.86798 | 11.39086 | 17.29642 | 3.57503 | 0.613556999 | low |
| TCGA-ZP-A9CZ | 1.934246575 | 0 | 4.110448 | 6.668995 | 18.46073 | 19.61178 | 19.645 | 7.647611 | 0.937561304 | high |
| TCGA-BC-A10Y | 1.947945205 | 1 | 7.590488 | 3.398666 | 15.35484 | 6.39678 | 11.12262 | 4.328907 | 0.560431704 | low |
| TCGA-DD-AAE9 | 1.978082192 | 0 | 2.853767 | 6.074594 | 15.8628 | 44.88287 | 27.50928 | 5.397454 | 0.992758933 | high |
| TCGA-DD-A73A | 1.994520548 | 0 | 1.271273 | 2.615541 | 10.77792 | 41.1252 | 9.763877 | 2.363329 | 0.502933276 | low |
| TCGA-FV-A2QQ | 1.997260274 | 0 | 1.456017 | 6.887016 | 12.39413 | 59.47469 | 21.6912 | 3.98501 | 0.947057996 | high |
| TCGA-G3-A5SK | 2.038356164 | 0 | 0.5377118 | 0.7691575 | 7.019687 | 9.029856 | 11.83003 | 2.291102 | 0.304187545 | low |
| TCGA-WX-AA46 | 2.071232877 | 0 | 1.803418 | 1.147325 | 8.46626 | 9.291401 | 11.09324 | 2.529132 | 0.3378864 | low |
| TCGA-2Y-A9GY | 2.073972603 | 1 | 4.870092 | 10.0916 | 17.86199 | 67.23952 | 58.74881 | 6.369158 | 1.643210435 | high |
| TCGA-DD-A4NJ | 2.082191781 | 0 | 8.946708 | 5.169147 | 15.34255 | 25.23803 | 15.03182 | 7.000592 | 0.805585978 | high |
| TCGA-DD-AAED | 2.090410959 | 0 | 2.169441 | 3.603383 | 14.27006 | 7.867779 | 59.52632 | 3.597914 | 1.05922963 | high |
| TCGA-G3-A5SI | 2.104109589 | 1 | 6.149339 | 4.436335 | 9.948623 | 11.09984 | 17.689 | 10.15712 | 0.76541925 | high |
| TCGA-G3-A3CH | 2.136986301 | 0 | 6.214072 | 2.978058 | 15.5245 | 19.10892 | 12.2896 | 5.37121 | 0.606857338 | low |
| TCGA-BC-A10T | 2.293150685 | 1 | 4.991267 | 6.345071 | 11.67071 | 16.59559 | 18.1916 | 7.715705 | 0.842130864 | high |
| TCGA-FV-A3I0 | 2.323287671 | 0 | 0.7234698 | 1.820703 | 12.423 | 11.06351 | 65.45171 | 7.235959 | 1.098218315 | high |
| TCGA-2Y-A9GZ | 2.323287671 | 1 | 2.772303 | 3.766329 | 9.521256 | 8.867871 | 12.37249 | 3.318288 | 0.510125171 | low |
| TCGA-GJ-A3OU | 2.408219178 | 0 | 2.560957 | 6.64641 | 11.38239 | 47.86873 | 31.8832 | 5.202814 | 1.033345875 | high |
| TCGA-XR-A8TG | 2.460273973 | 0 | 4.669647 | 2.94498 | 14.63468 | 28.12484 | 14.13538 | 5.296914 | 0.63639377 | low |
| TCGA-DD-A4NN | 2.463013699 | 1 | 7.031792 | 4.241332 | 16.18503 | 4.214097 | 16.64746 | 7.471726 | 0.726689447 | high |
| TCGA-KR-A7K8 | 2.482191781 | 0 | 2.045178 | 2.762114 | 9.125315 | 25.85035 | 12.63085 | 2.916442 | 0.497393695 | low |
| TCGA-DD-A1EJ | 2.753424658 | 1 | 7.190464 | 4.974029 | 26.58551 | 16.61975 | 40.40313 | 10.32017 | 1.208725141 | high |
| TCGA-DD-A4NA | 2.761643836 | 0 | 1.924002 | 8.55777 | 15.72917 | 21.69847 | 18.97918 | 6.827509 | 0.981582264 | high |
| TCGA-XR-A8TD | 2.821917808 | 0 | 3.442636 | 3.897335 | 14.07476 | 13.46094 | 23.17307 | 5.364114 | 0.732373969 | high |
| TCGA-DD-AAEK | 2.923287671 | 0 | 2.635417 | 1.754216 | 11.84608 | 20.66697 | 10.51363 | 4.740451 | 0.472812554 | low |
| TCGA-DD-A73F | 2.97260274 | 0 | 4.234536 | 4.089585 | 12.94824 | 9.170611 | 10.73341 | 4.178899 | 0.562254522 | low |
| TCGA-RG-A7D4 | 3.008219178 | 0 | 12.94668 | 7.487535 | 13.87133 | 29.70629 | 23.86685 | 8.424323 | 1.061493362 | high |
| TCGA-BC-A10Q | 3.109589041 | 1 | 1.219608 | 6.168094 | 24.98078 | 3.763149 | 31.25241 | 5.503492 | 0.995935801 | high |
| TCGA-DD-AADG | 3.136986301 | 0 | 3.475546 | 2.608142 | 9.115598 | 17.22348 | 13.24697 | 3.558466 | 0.490015387 | low |
| TCGA-DD-A114 | 3.147945205 | 1 | 8.39175 | 6.008904 | 19.19054 | 27.43832 | 24.96515 | 15.4936 | 1.16951136 | high |
| TCGA-DD-A4NK | 3.315068493 | 1 | 2.381047 | 2.115945 | 9.772347 | 13.21074 | 9.695723 | 2.19695 | 0.388780291 | low |
| TCGA-2Y-A9H1 | 3.367123288 | 1 | 1.101354 | 2.755186 | 5.995636 | 11.6368 | 10.26289 | 1.454469 | 0.369267164 | low |
| TCGA-DD-AADD | 3.37260274 | 0 | 8.220792 | 7.012662 | 18.48761 | 19.94683 | 25.52039 | 6.250633 | 1.006063409 | high |
| TCGA-DD-AADB | 3.402739726 | 0 | 4.817729 | 7.086868 | 19.83513 | 18.22767 | 32.34016 | 8.814802 | 1.131760706 | high |
| TCGA-2Y-A9GW | 3.482191781 | 1 | 1.868328 | 5.378406 | 9.288963 | 45.87225 | 14.57128 | 2.990119 | 0.708803622 | high |
| TCGA-DD-AACN | 3.567123288 | 0 | 2.418696 | 5.027051 | 16.86895 | 9.850579 | 17.9759 | 7.671291 | 0.789912811 | high |
| TCGA-XR-A8TC | 3.668493151 | 0 | 3.756582 | 5.07881 | 17.18692 | 15.7753 | 15.02604 | 13.23045 | 0.899939202 | high |
| TCGA-BC-A216 | 3.701369863 | 0 | 4.614219 | 4.995878 | 19.49402 | 8.190166 | 19.79727 | 4.513774 | 0.769078093 | high |
| TCGA-BD-A2L6 | 3.734246575 | 0 | 4.203396 | 1.857443 | 12.60803 | 5.52117 | 3.802436 | 2.671814 | 0.330637128 | low |
| TCGA-DD-A1EG | 3.75890411 | 1 | 2.471022 | 8.458378 | 14.11388 | 62.42652 | 30.26558 | 4.448009 | 1.155742749 | high |
| TCGA-DD-AACW | 3.901369863 | 0 | 6.655253 | 11.1217 | 12.3319 | 5.313778 | 27.66988 | 8.139618 | 1.1716456 | high |
| TCGA-2Y-A9H3 | 4.153424658 | 0 | 1.109285 | 1.698144 | 11.76075 | 4.939214 | 15.72057 | 1.688258 | 0.412217021 | low |
| TCGA-DD-AACV | 4.194520548 | 0 | 7.940882 | 1.71131 | 14.44351 | 12.38908 | 14.89806 | 5.369206 | 0.549700731 | low |
| TCGA-G3-A25T | 4.254794521 | 0 | 2.200624 | 8.313356 | 16.20849 | 4.531246 | 35.43393 | 5.359055 | 1.074542829 | high |
| TCGA-DD-A11D | 4.273972603 | 1 | 1.795194 | 1.328689 | 8.176661 | 12.0486 | 13.20268 | 3.844292 | 0.402531547 | low |
| TCGA-DD-AACU | 4.293150685 | 0 | 4.714987 | 3.706401 | 10.92366 | 32.38755 | 22.07149 | 5.287637 | 0.740787915 | high |
| TCGA-2Y-A9GT | 4.449315068 | 1 | 1.519528 | 1.786724 | 8.943046 | 8.707187 | 10.1305 | 2.8498 | 0.367302742 | low |
| TCGA-DD-AAW3 | 4.473972603 | 0 | 3.303694 | 3.596523 | 8.454348 | 15.15822 | 13.04577 | 4.03528 | 0.534555144 | low |
| TCGA-DD-AACC | 4.616438356 | 1 | 1.394389 | 2.762516 | 8.027388 | 6.702419 | 21.90388 | 3.527426 | 0.544269597 | low |
| TCGA-DD-A39X | 4.64109589 | 1 | 1.299439 | 11.04203 | 13.79735 | 31.08783 | 23.33711 | 2.124847 | 1.063491718 | high |
| TCGA-DD-AAVX | 4.706849315 | 0 | 1.381813 | 4.007088 | 8.4744 | 13.46074 | 16.66009 | 2.682776 | 0.554817585 | low |
| TCGA-DD-AAVZ | 5.205479452 | 0 | 4.531092 | 5.874076 | 15.23321 | 9.234982 | 19.7567 | 5.975479 | 0.808063929 | high |
| TCGA-DD-AAVY | 5.397260274 | 0 | 2.647952 | 3.299642 | 11.13721 | 59.56713 | 8.58914 | 4.46914 | 0.628930365 | low |
| TCGA-DD-A1EB | 5.526027397 | 0 | 1.258357 | 3.500028 | 8.633361 | 17.74929 | 10.25455 | 3.133336 | 0.482208861 | low |
| TCGA-DD-AADR | 5.556164384 | 0 | 3.953026 | 6.001528 | 11.0367 | 61.02592 | 24.89662 | 4.719159 | 0.954812746 | high |
| TCGA-DD-AACJ | 5.75890411 | 0 | 1.721297 | 6.101192 | 7.479516 | 42.38057 | 10.77175 | 2.683959 | 0.670571998 | low |
| TCGA-DD-AAVU | 6.032876712 | 0 | 2.136546 | 3.275421 | 9.062217 | 13.05401 | 24.47309 | 3.12377 | 0.619538127 | low |
| TCGA-DD-AACA | 6.304109589 | 0 | 3.526706 | 3.840679 | 10.15818 | 23.62428 | 13.02439 | 6.160476 | 0.630500866 | low |
| TCGA-DD-AACB | 6.367123288 | 0 | 4.663571 | 5.117831 | 14.47434 | 28.48513 | 21.04652 | 3.462555 | 0.781753932 | high |
| TCGA-ZS-A9CF | 6.608219178 | 0 | 5.172156 | 7.516574 | 9.351451 | 13.99225 | 7.925891 | 6.165871 | 0.729191032 | high |
| TCGA-FV-A4ZP | 6.810958904 | 1 | 4.758406 | 3.356825 | 12.93706 | 18.00929 | 29.79928 | 2.125683 | 0.718760474 | high |
| TCGA-2Y-A9GV | 6.936986301 | 1 | 1.673497 | 1.219635 | 10.46094 | 12.32498 | 13.38373 | 2.729085 | 0.396331966 | low |
| TCGA-DD-AAVP | 7.539726027 | 0 | 2.30121 | 3.029268 | 11.46427 | 10.59145 | 10.95849 | 5.557678 | 0.524529102 | low |
| TCGA-DD-A4NP | 8.504109589 | 0 | 1.2221 | 0.9446387 | 9.686151 | 5.287962 | 8.042194 | 2.078244 | 0.282178177 | low |
| TCGA-DD-A3A5 | 8.561643836 | 1 | 4.570878 | 3.014556 | 15.6633 | 10.11932 | 15.66673 | 6.513797 | 0.638574066 | low |
| TCGA-DD-A118 | 9.416438356 | 0 | 1.160621 | 6.084861 | 13.09459 | 14.92106 | 13.9098 | 3.328504 | 0.685393393 | low |
| TCGA-2Y-A9H0 | 10.06849315 | 0 | 12.50034 | 7.209901 | 17.74343 | 19.36689 | 36.03096 | 6.595873 | 1.145173218 | high |
