# Supplementary material for: Six Metabolism Related mRNAs Predict the Prognosis of Patients With Hepatocellular Carcinoma
Source: Front Mol Biosci. 2021 Feb 25;8:621232. doi: 10.3389/fmolb.2021.621232 (PMC8045485; doi:10.3389/fmolb.2021.621232)
Supplement: Supplementary file 1 [file datasheet1.zip › Supplementary tables/Supplementary table 5.docx]

Supplementary table 5: The Hazard ratio (HR), HR.95L and HR.95H value of each mRNAs

Training cohort (uniCox)

| id | HR | HR.95L | HR.95H | P value |
| --- | --- | --- | --- | --- |
| age | 0.9983483 | 0.9756921 | 1.0215306 | 0.8877574 |
| gender | 1.2115492 | 0.6237216 | 2.3533758 | 0.5710656 |
| grade | 1.0523048 | 0.6804092 | 1.6274695 | 0.8187435 |
| stage | 2.0066167 | 1.4363855 | 2.8032242 | 4.45E-05 |
| T | 1.9835885 | 1.4432783 | 2.7261708 | 2.43E-05 |
| M | 3.6344754 | 0.8701348 | 15.180879 | 0.0768528 |
| N | 4.7808337 | 0.6429244 | 35.550637 | 0.1264016 |
| riskScore | 7.4934041 | 3.8726742 | 14.49931 | 2.23E-09 |

| Training cohort (multiCox) | | |  |  |
| --- | --- | --- | --- | --- |
| id | HR | HR.95L | HR.95H | pvalue |
| age | 1.018423 | 0.9929019 | 1.0446 | 0.1585891 |
| gender | 1.2456598 | 0.5875774 | 2.6407898 | 0.5666657 |
| grade | 0.7495838 | 0.4421493 | 1.2707831 | 0.2845229 |
| stage | 2.1646892 | 0.451665 | 10.374679 | 0.3341002 |
| T | 0.8141242 | 0.1948603 | 3.4014011 | 0.7780295 |
| M | 1.5458463 | 0.2672419 | 8.9418643 | 0.6266882 |
| N | 2.3263285 | 0.2110329 | 25.644361 | 0.4905197 |
| riskScore | 9.559478 | 3.923856 | 23.289239 | 6.73E-07 |

| Validation cohort (uniCox) | | | | | |  | |  | |
| --- | --- | --- | --- | --- | --- | --- | --- | --- | --- |
| id | | HR | | HR.95L | | HR.95H | | pvalue | |
| age | | 1.0140259 | | 0.9852969 | | 1.0435925 | | 0.3421916 | |
| gender | | 0.4822423 | | 0.2434055 | | 0.9554329 | | 0.0365589 | |
| grade | | 0.9077466 | | 0.5735828 | | 1.4365911 | | 0.6794281 | |
| stage | | 1.7397455 | | 1.198318 | | 2.5258022 | | 0.0036018 | |
| T | | 1.661095 | | 1.1814366 | | 2.3354926 | | 0.0035116 | |
| M | | 4.0836572 | | 0.5496249 | | 30.341159 | | 0.1691197 | |
| N | | 1.2435964 | | 0.1681823 | | 9.1955692 | | 0.8308846 | |
| riskScore | | 5.243986 | | 2.2560192 | | 12.189342 | | 0.0001179 | |
| Validation cohort (multiCox) | | | | |  | |  | |  |
| id | HR | | HR.95L | | HR.95H | | pvalue | |  |
| age | 1.0213648 | | 0.9888478 | | 1.0549511 | | 0.2003358 | |  |
| gender | 0.4656171 | | 0.2140269 | | 1.0129531 | | 0.053916 | |  |
| grade | 0.8589376 | | 0.4895646 | | 1.5069999 | | 0.5960197 | |  |
| stage | 0.8295801 | | 0.1364253 | | 5.0445424 | | 0.8392446 | |  |
| T | 1.6490716 | | 0.3295203 | | 8.2527156 | | 0.5426449 | |  |
| M | 1.812512 | | 0.1819544 | | 18.055076 | | 0.6121029 | |  |
| N | 0.5589639 | | 0.0183381 | | 17.037803 | | 0.7386578 | |  |
| riskScore | 7.35155 | | 2.6487219 | | 20.404289 | | 0.0001281 | |  |
